# Co-Expression of Transcriptional Regulators and Housekeeping Genes in *Streptomyces* spp.: A Strategy to Optimize Metabolite Production

**DOI:** 10.3390/microorganisms11061585

**Published:** 2023-06-15

**Authors:** Lorena Cuervo, Mónica G. Malmierca, Raúl García-Salcedo, Carmen Méndez, José A. Salas, Carlos Olano, Ana Ceniceros

**Affiliations:** 1 Functional Biology Department, University of Oviedo, 33006 Oviedo, Spain; 2 University Institute of Oncology of Asturias (I.U.O.P.A.), University of Oviedo, 33006 Oviedo, Spain; 3 Health Research Institute of Asturias (ISPA), 33011 Oviedo, Spain

**Keywords:** *Streptomyces*, global regulators, overproduction, heterologous expression

## Abstract

The search for novel bioactive compounds to overcome resistance to current therapeutics has become of utmost importance. *Streptomyces* spp. are one of the main sources of bioactive compounds currently used in medicine. In this work, five different global transcriptional regulators and five housekeeping genes, known to induce the activation or overproduction of secondary metabolites in *Streptomyces coelicolor*, were cloned in two separated constructs and expressed in 12 different strains of *Streptomyces* spp. from the in-house CS collection. These recombinant plasmids were also inserted into streptomycin and rifampicin resistant *Streptomyces* strains (mutations known to enhance secondary metabolism in *Streptomyces*). Different media with diverse carbon and nitrogen sources were selected to assess the strains’ metabolite production. Cultures were then extracted with different organic solvents and analysed to search for changes in their production profiles. An overproduction of metabolites already known to be produced by the biosynthesis wild-type strains was observed such as germicidin by CS113, collismycins by CS149 and CS014, or colibrimycins by CS147. Additionally, the activation of some compounds such as alteramides in CS090a pSETxkBMRRH and CS065a pSETxkDCABA or inhibition of the biosynthesis of chromomycins in CS065a in pSETxkDCABA when grown in SM10 was demonstrated. Therefore, these genetic constructs are a relatively simple tool to manipulate *Streptomyces* metabolism and explore their wide secondary metabolites production potential.

## 1. Introduction

The current growing resistance to antibiotics due to the misuse of drugs, as well as their abusive use in livestock, threatens to be one of the main causes of death in the near future. The SARS-CoV-2 pandemic has culminated, among many other effects, in the acceleration of antibiotic resistance due to antibiotic prescriptions given to SARS-CoV-2-infected patients to prevent secondary bacterial infections [1,2]. It is urgent to find new treatments against new diseases as well as to improve existing ones. Different strategies are followed to avoid resistance mechanisms, such as combining different compounds that have a synergic activity, chemical modification of current compounds to alter their activity, looking for novel bioactive compounds produced by microorganisms isolated from underexplored environments, or the use of bacteriophages, among others [3,4,5,6,7,8,9].

Natural products have long provided the active principle for many drugs due to their enormous structural and chemical diversity. However, after the golden era of drug discovery in the 1950s, it is increasingly challenging to find new metabolites of interest. Improvement in sequencing techniques and bioinformatics analysis has shown that microorganisms have the potential to produce many unknown secondary metabolites that could potentially have novel structures, which could avoid current resistance mechanisms [8,9,10,11]. However, despite the wide array of -omic technologies currently available, the continuous rediscovery of already known compounds together with the technical limitations for detecting compounds is increasing the difficulties of finding novel products. Furthermore, it is not always possible to obtain a sufficient level of production by the microorganism to make the compound attractive for industrial-scale manufacturing. Many compounds are produced under very specific conditions, which are difficult to reproduce in the laboratory. On some occasions, the gene clusters responsible for the biosynthesis of these metabolites might not be complete or an unknown regulator is repressing its expression [12,13]. In other cases, precursors might not be available for biosynthesis or are being exhausted by other biosynthesis pathways. Many strategies have been developed to overcome these problems, such as redirecting precursors to the target biosynthesis by disrupting or deleting highly active routes that may be consuming precursors, testing different production media and conditions, heterologous expressing genes that seem to be missing or defective, or regulators expected to activate the pathways [12,13,14,15].

There are many successful examples of transcriptional regulator engineering applied to the activation of secondary metabolites. In 2018, Guo and co-workers improved avermectin production by *S. avermitilis* by deciphering the regulatory cascade involving the SAV4189 pathway-specific transcriptional activator [16]. However, the engineering of global regulators can also be used to modify the production of metabolites of interest, as was demonstrated by the activation of nikkomycin biosynthesis by the disruption of the *adpA* gene in *Streptomyces ansochromogenes* [17]. Previous studies have shown how the introduction or deletion of several regulators at the same time generates different synergistic effects on the recipient strain [18].

Ribosome engineering is a strategy that relies on the discovery of strains with mutations in their ribosome or RNA polymerase (through screening of streptomycin and rifampicin resistant mutants), resulting in the enhancement of enzyme production. Analysis of the metabolite profile of these resistant strains showed the production of metabolites that are undetectable in wild-type strains [19,20].

As mentioned above, one interesting approach for new drug discovery is to explore the biosynthetic potential of microorganisms that live in underexplored ecosystems or symbiosis with other organisms. Insect microbiota has recently drawn attention for its antifungal and antimicrobial activities [17,18]. The Carlos Sialer collection (CS), isolated from the tegument of ants from the Attini tribe, was recently analysed as a source of novel secondary metabolites [19,20].

In this work, we followed a multi-angle strategy for the modification of the secondary metabolism of twelve *Streptomyces* strains from the CS collection. Two different vectors were generated and introduced in the wild-type CS strains: one containing five housekeeping genes (pSETxkBMRRH) and another containing five regulatory genes (pSETxkDCABA). These genes were carefully selected based on their characteristics, and their involvement in the regulation of secondary metabolism and the production of bioactive compounds (Table 1 and Table 2). It was also estimated that the introduction of a battery of genes will generate a response that may not be generated with the introduction of these genes separately, since it is well known that regulators interact with each other [21,22,23,24].

In addition, streptomycin and rifampicin resistant strains were generated and their metabolic profiles were analysed after the introduction of the aforementioned vectors. This work aims to evaluate the effect of these housekeeping and global regulators genes when heterologously expressed in different *Streptomyces* strains.

## 2. Materials and Methods

### 2.1. Strains and Culture Media

The non-methylating strain of *Escherichia coli* ET12567 carrying pUZ8002 was used for conjugation with *Streptomyces*, as described in Kieser et al. [40]. Additionally, 2xTY (tryptone yeast) (16 g tryptone, 5 g NaCl, 10 g yeast extract per L) with appropriate antibiotics were used to grow *Escherichia coli* ET12567 carrying pUZ8002. SFM (soya flour mannitol) [40] with 0.1 mM MgCl_2_ was used for conjugation, as described in Kieser et al. [40]. *Streptomyces* from the CS collection strains (CSs) were grown in SFM and MA (A Medium) [41] for sporulation. The strains were kept as spores in 50% glycerol at −20 °C. For secondary metabolite production, strains were grown in five different production media: SM10 [42], R5A [43], SM17 (composition per litre: glucose 2 g, glycerol 40 g, soluble starch 2 g, Arkasoy (soy protein) 5 g, peptone 5 g, yeast extract 5 g, NaCl 5 g, CaCO_3_ 2 g, tap water), SM20 [44], and YEME-S (yeast extract-malt extract without sucrose) [40] containing different sources of carbon or nitrogen. *Streptomyces* strains used in this study are listed in Table S1 in Supplementary material.

### 2.2. Gene Selection

Genes selected for this study were carefully chosen based on the available bibliographical information showing the role of each gene in the metabolism of different *Streptomyces* species and, when available, the effect that their overexpression and/or deletion has on their secondary metabolites production (Table 1 and Table 2). In the case of *rpoB*, point mutations are known to activate secondary metabolism [39]. However, this gene was selected for overexpression in an effort to improve the quantity of available RNA polymerase in the cell and, consequently, to reinforce the transcriptional machinery.

### 2.3. pSETxk Construction

**Figure 1:**
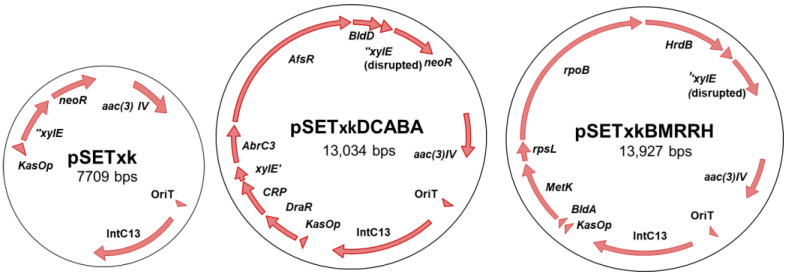
Schema of the constructs used in this work. From left to right, pSETxk, pSETxkDCABA, and pSETxkBMRRH. pSETxkDCABA and pSETxkBMRRH have fragmented *xylE* since an enzyme has been used that has disrupted this gene, which is not necessary for this experiment.

To construct plasmid pSETxk, the kasOp* constitutive promoter was extracted from plasmid pDR4-K* [36] by digestion with BamHI/SpeI. The released DNA fragment was blunt-ended by T4 DNA polymerase and cloned in the PstI site (blunt-ended) of plasmid pOJ260 [45]. The correct orientation of the promoter relative to the multiple cloning site in the resulting plasmid (pOJ260k) was confirmed by PCR with primers dKAS-check and rvKAS-check. Then, a 1.8kb-DNA fragment containing genes *xylE* and *neoR* was amplified by PCR from pDR4-K* with primers SmaI-NsiI-REP and MunI-REP, and cloned in the EcoRV/EcoRI sites of pOJ260k to afford plasmid pOJk-REP. Finally, a 2.1kb-DNA stretch encompassing kasOp*, xylE, and neoR was amplified from pOJk-REP with primers BglII-KasOd and MunI-REP and inserted into the BamHI/EcoRI sites of plasmid pSET152 [45] to produce pSETxk (Figure 1). (Primers are summarized in Table S2, Supplementary Material).

### 2.4. Construction of a Multiregulator Recombinant Plasmid

Genes were amplified from *Streptomyces coelicolor* chromosome using primers shown in Table S2 in Supplementary material. Primers were designed to amplify each gene with an individual restriction site at each side to facilitate directed and consecutive cloning of the genes. All genes were cloned in a single operon controlled by the strong constitutive promoter *kasOp*, with the promoterless gene *neoR* at the end of the operon, serving as an indication that the expression of all genes is correct when the recombinant strains were resistant to kanamycin. The amplification of each gene was designed in such a way that includes the native RBS (ribosomal binding site) and avoids the inclusion of the transcriptional terminator that would stop the expression of the artificial operon. The EcoRV restriction site was needed to be able to clone all the genes, which truncated the *xilE* gene that was not necessary for this work.

The final constructs were designated pSETxkDCABA, which contains the genes *draR*, *CRP* (cyclic AMP receptor protein*)*, *abrC3*, *bldD* and *afsR*, and pSETxkBMRRH, which contains *bldA*, *metK* (methionine adenosyltransferase), *rpsL* (30S ribosomal protein S12), *rpoB* (RNA polymerase subunit beta), and *hrdB* (Figure 1).

### 2.5. Strain Construction

PSETxkDCABA, pSETxkBMRRH, and the empty vector pSETxk were introduced in wild-type strains of *Streptomyces* through conjugation, following the protocol in Keiser et al. [40]. To verify whether each strain contained all the genes cloned in each construct, the genomic DNA of each strain was extracted. PCR amplifications were performed on at least two contiguous regulators, using the free constructs as a positive control. Furthermore, resistance to apramycin and kanamycin was confirmed. The primers used for strain confirmation are listed in Table S3 Supplementary material.

### 2.6. Generation of Spontaneous Mutants Resistant to Rifampicin and Streptomycin

Wild-type strains were cultured on MA supplemented with 50 µg/mL streptomycin or 100 µg/mL rifampicin to obtain spontaneous single mutants [46,47]. Subsequently, they were cultured on MA with 100 µg/mL streptomycin or 200 µg/mL rifampicin to verify the acquisition of resistance. pSETxkDCABA, pSETxkBMRRH, and the empty vector pSETxk were also introduced in these strains.

### 2.7. Production of Secondary Metabolites in Liquid Media

Spores from each strain were used to inoculate the precultures in TSB (tryptic soy broth), using baffled flasks for proper dispersion of mycelia. In total, 50 mL of each media were inoculated from the precultures, with an initial O.D. of 0.2. Cultures were grown for 13 days at 250 rpm and 30 °C. Whole culture samples were taken after 4, 6, 8, and 13 days. The samples were extracted with three different organic solvents: ethyl acetate, acidic ethyl acetate (1% formic acid), and butanol. Samples were left to mix with the solvent for 1–2 h of shaking. The solvent phase was then separated by centrifugation and dried under vacuum (Labcono CentriVap Benchtop Vacuum Concentrator). For each set of strains and media (for example, CS014 wild-type, and recombinant strains from this parent strain in SM10), the sample with the highest dry weight was resuspended in 100 µL of methanol and the rest of the samples were resuspended in a proportional volume to the mass of the dry weight to keep a similar concentration for all samples. Then, 10 µL were injected into the UPLC and LC/MS for analysis. The amount of methanol added to the samples was normalized to the dry weight of mycelia.

### 2.8. Secondary Metabolites Production on Solid Media

Spores were plated on R5A agar and YEME-S agar for 5 days [48]. This modified version of YEME-S (without sucrose) was used to limit the available carbon source and it is useful for the analysis of growth and antifungal production. Then, 3.5 g of each culture was extracted with three different solvents: ethyl acetate, acidic ethyl acetate (1% formic acid), and butanol. After 1–2 h of mixing with solvents, the organic phase was collected and evaporated in the same way as described above. Subsequently, the dried extracts with the highest dry weights were resuspended in 100 µL of methanol and the rest of the samples were resuspended in a proportional volume to the mass of the dry weight to keep a similar concentration for all samples. Then, 10 µL of samples were injected into the UPLC.

### 2.9. Bioactivity Analysis from Solid-Media Samples

Agar diffusion bioassays against *Micrococcus luteus* (Gram-positive), *Escherichia coli* (Gram-negative), and the yeast *Candida albicans* were performed to test for the antibiotic production. TSA (agar tryptic soy both) were used for *M. luteus* and *E. coli* assays and YMA (yeast extract 3 g; malt extract 3 g; peptone 5 g; and glucose 10 g per litre) was used for *C. albicans* assays. Two different bioassays were performed: (i) a 6 mm agar plug from each actinobacteria culture (grown as described in 2.8 section) was placed on top of the bioassay plate; and (ii) solvent-extracted samples were resuspended on methanol and 20 µL of each sample was added into a diffusion bioassay disc. The plates were then incubated at 4 °C for one hour to allow the metabolites to diffuse into the surrounding medium and finally incubated for 16 h at 30 °C (antifungal tests) or 37 °C (antibacterial tests). The diameter of the inhibition zones was measured and compared with the control sample. Each test was performed in triplicate.

### 2.10. Chromatographic Analysis

Samples were run on an Acquity UPLC I-Class (Waters, Mildford, MA, USA) using a BEH C18 column (1.7 μm particle size, 2.1 mm × 100 mm) and acetonitrile and water containing 0.1% of trifluoroacetic acid as mobile phase. A gradient was used from 10 to 99% of acetonitrile in 10 min and a flow rate of 0.5 mL/min. For HPLC/MS analysis, a Waters ZQ4000 system was used connected to an HPLC 2695/2795 (An Alliance chromatographic system coupled to a SunFire C18 column (3.5 μm particle size, 2.1 mm × 150 mm) and a 996 PDA detector. Acetonitrile and MQ water + formic acid 0.1% were used as the mobile phase and elution was performed with an isocratic hold with acetonitrile (10%) for 4 min followed by a linear gradient of acetonitrile (10–88%) over 30 min (0.25 mL/min). Mass analysis was performed by ESI (electrospray ionization) in the positive mode with a capillary voltage of 3 kV and cone voltage of 20 kV. The Empower 3.0 program was used to compare and analyse the chromatograms obtained from each sample.

### 2.11. Prediction of Secondary Metabolites Biosynthetic Gene Clusters

Web-based software antiSMASH 7.0 was used to analyse the genomic sequence of these strains to detect the putative secondary metabolites gene clusters present in their chromosomes [49,50]. It was considered that the prediction is accurate when the percentage of identity is greater than 85%.

### 2.12. Dereplication Assay

HRMS-based compound dereplication was performed at Medina Foundation. The in-house library and the Dictionary of Natural Products version 26:2 were used to identify already known compounds. LC-MS was performed on Agilent 1200 Rapid Resolution HPLC. Analysis was performed on a maXis Bruker qTOF mass spectrometer. The volume injected was two µL and a Zorbax SB-C8 column (2.1 × 30 mm, 3.5 µm particle size) was used for the separation. The mobile phase consisted of solvent A, 90:10 milliQ water-acetonitrile, and solvent B, milliQ water-acetonitrile, both with 13 mM ammonium formate and 0.01 TFA. Samples were eluted with a 0.3 mL/min flow rate, and the gradient used was 90% to 0% solvent A/10% to 100% solvent B in 6 min, 0% solvent A/100% solvent B in 2 min, 0% to 90% solvent A/10% to 100% solvent B in 0.1 min, and 90% solvent A/10% solvent B for 9.1 min. The maXisqTOF mass spectrometer was operated in ESI positive mode. Source conditions were 4 kV capillary voltage, end plate offset = 500 V, dry gas (N2) flow = 11 L/min; dry temperature = 200 °C, and nebulizer (N2) pressure at 2.8 bars. The retention time, together with the exact mass and the derived molecular formula, was used as the criteria to search in databases.

## 3. Results and Discussion

Five different pleiotropic regulators (Table 1) and five different housekeeping genes (Table 2) were selected to be overexpressed in *Streptomyces* sp. strains by the constructions pSETxkDCABA and pSETxkBMRRH, respectively (Figure 1). These regulators were selected based on bibliographic references where higher production of one or more of the known antibiotics from *Streptomyces* spp. was induced by the overexpression of these genes. We also selected pleiotropic genes that have shown that their overexpression or point mutations in their sequence induced a higher production of secondary metabolites in *Streptomyces* spp. Previous studies have shown how the introduction or deletion of several regulators at the same time generates different synergistic actions and that global regulators interact with each other [18,22]. The introduction of a panel of genes will, therefore, generate a response that may not be generated with the introduction of these genes separately.

pSETxkDCABA and pSETxkBMRRH were introduced in twelve different strains of *Streptomyces* obtained from the CS strain collection [42] and in rifampicin and streptomycin resistant strains in order to modify their secondary metabolism. Rifampicin resistant mutants contain mutations in the *rpoB* gene that codes for RNA polymerase, and streptomycin resistance is accomplished by mutations in the *rpsL* gene that codes for the ribosomal protein S12. Both mutations are known to induce the production of secondary metabolites [51,52,53]. It was, therefore, expected that the introduction of pSETxkBMRRH or pSETxkDCABA in these resistant strains, which already possess a modified secondary metabolism, would have a different effect than in the wild-type strains. These strains were then grown in different media containing different carbon and nitrogen sources. Whole-culture samples were extracted with three different organic solvents and extracts were then analysed by UPLC. The chromatographic profiles of the extracts were compared with those of the wild-type strain and the control of each strain containing the empty vector, grown under the same conditions. When a different production profile was found, a dereplication analysis was conducted to discriminate between putative novel compounds from the already described ones. By applying this strategy, it was possible to detect the overproduction of some already known compounds and the activation of new ones. Then, the metabolic potential of each strain was analysed using antiSMASH to determine which secondary metabolites are predicted to be produced [54]. Thus, it is intended to determine a production optimization strategy to make it more efficient at an industrial level, as well as to identify new compounds. In the current era, both due to the growing increase in antibiotic resistance as well as the constant rediscovery of the same compounds, it is necessary to apply different approaches to discover new drugs and improve the efficiency of production of those already known [3]. The effects of these constructs on the metabolism of the different strains of *Streptomyces* spp. observed by chromatographic analysis were classified into five categories: overproduction of compounds, activation of the production, inhibition of the production, modification of the production as an effect of the insertion of the empty vector, and overproduction in spontaneous rifampicin/streptomycin resistant strains.

### 3.1. Modification of the Production Profile as an Effect of Insertion of the Empty Vector

**Figure 2:**
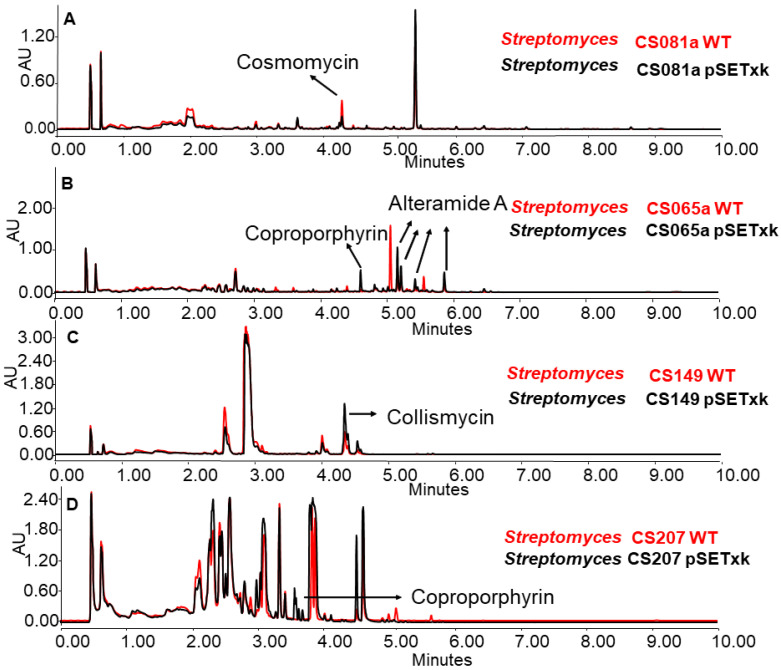
Comparative UPLC analysis of samples of the wild-type strain (in red colour) and the control strain carrying the empty vector (in black colour). (**A**) CS081a cultured in liquid SM10 medium at day 13 of culture and extracted with ethyl acetate. (**B**) CS065a in liquid SM20 at day 8 and extracted with ethyl acetate. (**C**) CS149 in liquid R5A at day 13 and extracted with ethyl acetate and CS207 in liquid. (**D**) CS207 in liquid SM17 at day 4 and extracted with butanol.

In several cases, it was observed that the production of some secondary metabolites was reduced, increased, or even activated as a consequence of the insertion of pSETxk into the chromosome of the different CS strains. It has already been shown that the introduction of an empty vector in a bacterial strain can have an effect on its secondary metabolism [55]. Thus, Figure 2 shows some examples of how the insertion of the empty vector caused a metabolic change that led to the increased production of compounds, as is the case for coproporphyrins and alteramides in CS065a, coproporphyrins in CS207 (Figure 2B,D), or collismycins in CS149 (Figure 2D). Additionally, it caused a decrease in the production of different compounds, as is the case for cosmomycins in CS081a. In the rest of the analyses, the wild-type strain and the empty vector strains showed identical production profiles. 

### 3.2. Overproduction of Secondary Metabolites

**Figure 3:**
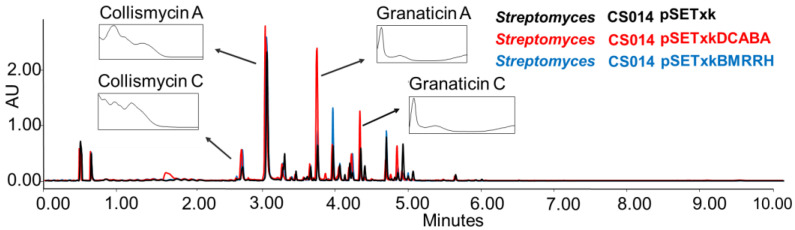
Comparative UPLC analysis of CS014 samples cultured in liquid SM10 medium after four days of growth and extraction with ethyl acetate. The ultraviolet-visible (UV-vis) spectra of the detected collismycin and granaticin compounds are shown.

In most strains, we could observe an overproduction of compounds. Some of these compounds were identified by a combination of techniques. First, a bioinformatic analysis of each sequence was performed using antiSMASH, which predicted the putative biosynthetic gene clusters harboured in each chromosome. Then, HPLC and MS analysis of the extracts together with dereplication allowed us to identify compounds produced in different media by each strain. The most relevant results are shown in Table 3:

CS014: this strain is known to produce granaticin, a coloured antibiotic from the benzoisochromanequinone family polyketide, to which actinorhodin belongs too, and collismycins antibiotics [56]. When the recombinant plasmids constructed for this work were inserted in this strain, an overproduction was observed of both compounds in all of the tested conditions (Figure 3).

**Figure 4:**
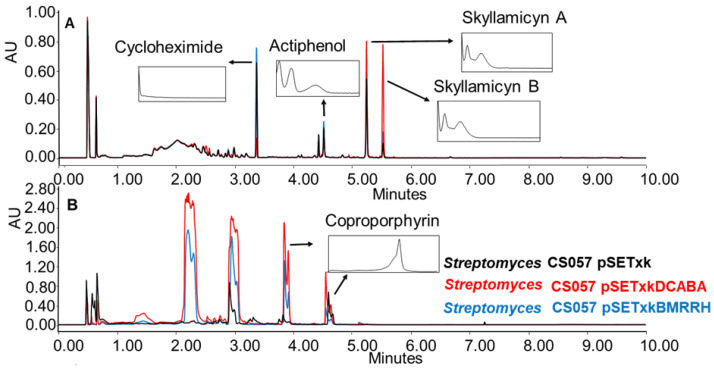
(**A**) Comparative UPLC analysis of CS057 samples cultured in liquid R5A medium at day 8 of culture and extracted with ethyl acetate. (**B**) Comparative UPLC analysis of samples of CS057 cultured in liquid SM10 medium at day 8 of culture and extracted with ethyl acetate with 1% formic acid. UV-vis spectra of the detected compounds are shown.

CS057: This strain is known to produce the strong inhibitors of platelet-derived growth factor skyllamycin A and B [57], actiphenol, and cycloheximide, which both inhibit eukaryotic translation [58,59]. All three compounds have been overproduced by both recombinants in R5A, SM17, and SM10 media. Furthermore, the production of coproporphyrin, a metal chelate [60,61], was activated in SM10 and SM20 media (Figure 4).

**Figure 5:**
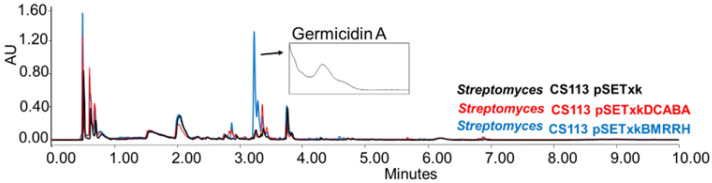
Comparative UPLC analysis of CS113 samples cultured in liquid R5A medium at day 8 of culture and extracted with ethyl acetate. UV-Vis spectrum of germicidin A shown.

CS113: When carrying the construct pSETxkBMRRH, this strain overproduced germicidin, a germination-inhibitor compound A [62,63] when cultured in R5A and SM10 media and extracted with any of the solvents tested (Figure 5).

**Figure 6:**
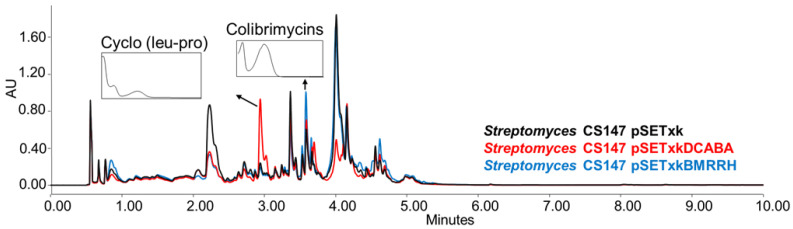
Comparative UPLC analysis of CS147 samples cultured in liquid R5A medium at day 4 of culture and extracted with ethyl acetate with 1% formic acid. UV-vis spectrum of cyclo (Leu-Pro) and colibrimycins are shown.

CS147: In all of the media tested, both mutants overproduced the antibiotic Cyclo (leu-pro), colibrimycins (a hybrid polyketide synthase-nonribosomal peptide synthetase only detected when cultures were extracted with ethyl acetate containing formic acid or butanol) and N-acetyltyramine (with antimicrobial properties,) (Figure 6) [64,65,66,67]. Additionally, in SM17 and SM10, the overproduction of coproporphyrins could be observed by both recombinants. In R5A, N-chloroacetyl tryptophan was overproduced by both recombinants when cultures were extracted with butanol.

**Figure 7:**
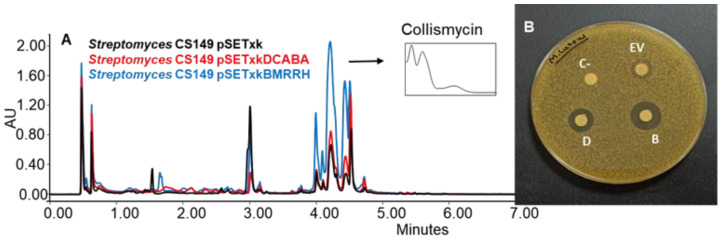
(**A**) Comparative UPLC analysis of C149 samples cultured on agar R5A medium and extracted with butanol. UV-vis spectrum of collismycinis shown. (**B**) Bioassay of the samples extracted with butanol from solid R5A and resuspended in methanol against *M. luteus.* In total, 20 µL methanol was used as negative control (C-). EV indicates where the sample from CS149 pSETxk was spotted; D indicates the sample of CS149 pSETxkDCABA; and B the sample of CS149 pSETxkBMRRH.

CS149: CS149 bearing pSETxkBMRRH showed an overproduction of collismycins in all media tested. Coproporphyrins were also overproduced in SM17 and SM10 media by both recombinants. Bioassay of the extracts obtained from the culture on agar R5A (with the three solvents, ethyl acetate, ethyl acetate with 1% formic acid, and butanol) of the recombinants of this strain showed differential bioactivity against *M. luteus.* The empty vector control generated a growth inhibition zone of 11 mm of diameter in the bioassay, pSETxkDCABA a halo of 20 mm of diameter, and pSETxkBMRRH a halo of 24 mm of diameter (Figure 7). This activity may be due to the distinctive production of collismycin, although the height of the peaks from the empty vector control and the strain carrying pSETxkDCABA is quite similar while the size of the inhibition halo is almost double the one observed in the empty vector control, which could mean that the bioactivity observed is caused by a different compound not detected by UPLC.

All of the results shown in this work are a summary of an exhaustive screening of metabolite production on solid and in liquid media and the application of various methods of extraction, with the aim of analysing the production of secondary metabolites. The metabolic profiles of the different recombinant strains compared to the controls (wild-type and the strain containing the empty vector), revealed a general increase in the production of compounds, many of which could be identified by dereplication. The data corresponding to the identification of compounds whose production was activated/overproduced in this work are shown in Supplementary data. In all strains, the increased production of compounds was shown by at least one of the genetic constructs introduced. However, the increase was not equally efficient in all strains or for all compounds they produce; it was influenced by the test conditions (culture medium and extraction method). For each strain and compound, it is necessary to determine the ideal production conditions to observe the effect of the regulators inserted with greater efficiency.

### 3.3. Activation of Secondary Metabolites

**Figure 8:**
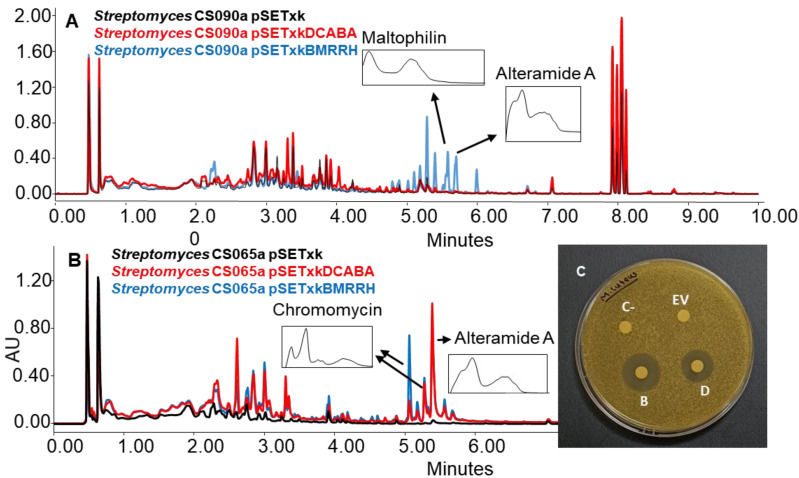
(**A**) Comparative UPLC analysis of samples of CS090a cultured on agar R5A medium and extracted with ethyl acetate with 1% formic acid. (**B**) UPLC analysis of samples of CS065a culture on agar YEME-S medium and extracted with ethyl acetate with 1% formic acid. UV-vis spectra of alteramide and chromomycin are shown. (**C**) Bioassay plate against M. luteus of the samples extracted with ethyl acetate with 1% formic acid and resuspended in methanol. In total, 20 µL of methanol was used as negative control (C-). EV indicates where the sample of the culture from CS65a pSETxk was assayed; D indicates where the sample from CS065a pSETxkDCABA was spotted. B shows where the sample from CS065a pSETxkBMRRH was assayed.

The strains bearing pSETxkDCABA and pSETxkBMRRH showed, on some occasions, the activation of different metabolic pathways. Specifically, the activation of the synthesis of alteramides was observed for the CS090a pSETxkBMRRH strain cultured in R5A, while the strain containing the empty plasmid did not produce this compound or did in undetectable levels. It also overproduced maltophilins (Figure 8A). Similarly, this effect was observed in the CS065a strain where both recombinants overproduced alteramides and the production of chromomycins got activated when cultured in YEME-S medium. Extracts from these cultures showed a growth inhibition against M. luteus when tested in a bioassay. pSETxkDCABA produced an 11 mm diameter halo and pSETxkBMRRH produced a 20 mm halo. The negative control or the strain containing the empty vector did not produce any growth inhibition (Figure 8B). Presumably, this bioactivity is due to chromomycin production, since lower production was observed in CS065a pSETxkDCABA than CS065a pSETxkBMRRH, which correlates with the halo size observed from both strains (Figure 8B,C).

The de novo biosynthesis of alteramides and chromomycins by these mutants in YEME-S manifests the great potential of this genetic approach to awaken silent biosynthetic gene clusters that govern the production of bioactive compounds which may be inactive in tested conditions. An interesting observation is that the same strains grown in R5A liquid media did not show the same production as in R5A solid media, stating the importance of performing a screening with different settings conditions to determine the best production conditions for each strain and compound.

In most of the cases shown, some of the overproduced or activated metabolites were identified. Compounds that remained unidentified were either produced in too small amounts to be able to obtain a proper identification or they are not present in the library used for the dereplication. Further optimization of their production would be needed, and isolation of the compounds followed by structural elucidation would be needed to know their structure and be able to test their bioactivity.

### 3.4. Inhibition of the Secondary Metabolite Production

**Figure 9:**
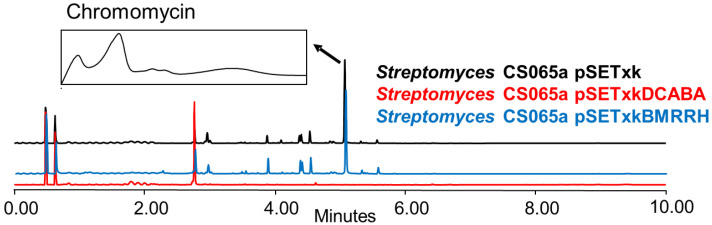
Comparative UPLC analysis of samples of CS065a cultured in liquid SM10 medium at day 4 of culture and extracted with ethyl acetate. UV-vis spectrum of chromomycin is shown.

Global regulators, also known as pleiotropic regulators, up-regulate and down-regulate the primary and secondary metabolism of bacteria [21]. In some cases, these changes result in activation or overproduction, as described in the previous sections. In other cases, they can repress or down-regulate the expression of genes that result in the inhibition of the production of other compounds [26]. From all strains analysed, we only observed one case of inhibition which was in Streptomyces CS065a bearing pSETxkDCABA and grown in SM10 media (Figure 9).

The inhibition of chromomycin biosynthesis might be a consequence of a deregulation effect on metabolism that, in this case, represses the biosynthesis pathway of this compound under very specific conditions. This result is exceptional since the genetic constructs used in the present study in most cases showed an increase in the production of compounds and not their inhibition. However, the alteration of metabolism can result in activation, overexpression, or inhibition depending on the strain or media. These effects can also be appreciated to a greater or lesser extent depending on the organic solvent used since compounds are extracted differently using different solvents, hence showing the importance of testing each strain under different conditions to obtain the desirable effect. The inhibition of chromomycins was only observed in SM10 medium when the Streptomyces CS065a contained the construct pSETxkDCABA; in the rest of the cases, the strain is capable of producing them and their production is considerably increased (Figure 8).

### 3.5. Effect of Spontaneous Rifampicin and Streptomycin Resistant Mutations on Secondary Metabolism

pSETxkDCABA and pSETxkBMRRH were also introduced into spontaneous rifampicin (R.R.) and streptomycin (R.S.) resistant strains that were obtained as described in the Materials and Methods section. Resistance to rifampicin and streptomycin has been shown to induce changes in the metabolism by improving the level of production of bioactive compounds and activating new metabolite synthesis [53]. Rifampicin resistant mutant strains contain mutations in their RNA polymerase rpoB gene while streptomycin resistant mutants have mutations in the S12 ribosomal protein rpsL [51,52,53]. Strains carrying pSETxkBMRRH, apart from their mutated rpsL and rpoB genes, contain the native genes from S. coelicolor. However, the mutations were not complemented, as the strains remained resistant to rifampicin or streptomycin.

**Figure 10:**
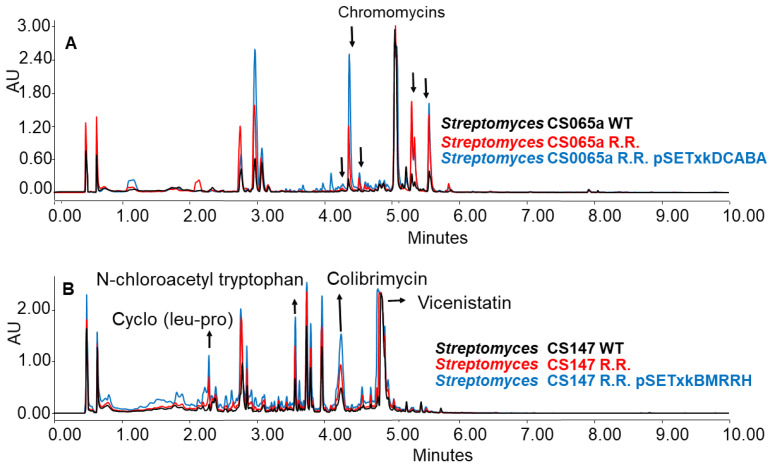
(**A**) Comparative UPLC analysis of samples from CS065a WT, CS065a R.R., and CS065a R.R. containing pSETxkDCABA cultured in liquid R5A medium at day 4 of culture and extracted with ethyl acetate. (**B**) Comparative UPLC analysis of samples from CS147 WT, CS147 R.R., and CS0147 R.R. containing pSETxkDCABA cultured in R5A medium at day 4 of culture and extracted with butanol.

The generated mutants showed an incremented metabolic profile compared to the wild-type strain. As shown in Figure 10 as an example, the generation of spontaneous resistance increased the production of chromomycins, Cyclo (leu-pro), N-chloroacetyl tryptophan, colibrimycins, vicenistatin, and other compounds that could not be identified. As in previous results, this increase is dependent on the culture media and solvents employed, thus varying the metabolic profile. Moreover, the introduction of the constructs contributes substantially to a further increase in production, again, depending on the media and solvent used in the extraction.

When CS065a R.R was cultured in R5A, chromomycins were overproduced. This overproduction was further increased by the insertion of any of the constructions, especially CS065a R.R. pSETxkDCABA (Figure 10A). Similarly, when CS147 was cultured in R5A, N-chloroacetyl tryptophan, colibrimycin, and cyclo (pro-leu) were overproduced. A slight improvement in vicenistatin production by CS147 R.R. pSETxkBMRRH can be seen (Figure 10B). However, the recombinant carrying pSETxkDCABA does not show a substantial production increase.

Furthermore, CS147 streptomycin resistant mutants have a generally increased production level compared to the wild-type strain. However, when both genetic constructs were introduced into the CS147 R.S. strain, the production was not increased under the conditions tested, although the CS147 strains containing both constructs had a better general production of compounds than the wild strain (Figure 10). This fact makes us consider that this regulator strategy can be much more efficient than the acquisition of spontaneous resistance, adding that the genes responsible for such an effect are known when introducing the constructs described in this work, while it is uncertain where spontaneous point mutations are located.

This attempt to improve secondary metabolism highlights the possibility of combining the insertion of global regulators and housekeeping genes with other classical techniques to improve compound production. This does not only imply greater profitability at the industrial level, but it is also an interesting tool that can be used in research, as many cryptic biosynthetic pathways remain unknown due to difficulties in activating them or because they are produced at such low levels that it is very difficult to detect them. The activation of metabolic pathways by these techniques or the increase in the production of a compound that is already intrinsically produced by the wild-type strain offers an advantage that can lead to improving the research and discovery of new natural products. Therefore, the simultaneous use of different techniques can be an efficient strategy to consider. The main advantage offered by the constructs described in this work is that their insertion into the chromosome is highly efficient, since the use of pSETxk, an integrative plasmid, makes it relatively easy to obtain recombinants with the increased production of secondary metabolites. On the other hand, ribosomal engineering is also another classic strategy that is easy to use to increase production [51,52,53]. The combination of these two approaches is just one example of the different technologies with which the use of global regulatory elements and housekeeping genes can be combined. In the case of rifampicin resistant mutants, results of increased production were obtained once the pSETxkDCABA or pSETxkBMRRH constructs were introduced. However, the insertion of the corresponding constructs in the streptomycin resistant mutants did not improve production under the tested conditions.

CRP, DraR, AbrC3, BldD, and sigma factor HrdB bind to promoters of structural genes or pathway specific regulators of different secondary metabolite biosynthetic clusters. These promoters must contain the corresponding consensus sequences so that these regulators can bind the DNA [25,31,32,68,69]. In the case of AfsR, MetK, BldA, RpoB, and RpsL, they induce the overproduction of compounds but in an indirect way. AfsK activates itself by autophosphorylating after binding to S-adenosyl-L-methyonine (SAM). Then, it phosphorylates AfsR which activates the transcription of afsS [70]. The mechanism of action of protein AfsS is not known, but it interacts with pathway specific regulators of secondary metabolites [22]. An overexpression in MetK results in high levels of S-adenosyl-L-methyonine, which is a methyl donor and has been known to directly activate transcriptional factors of antibiotic production [33]. The bldA gene is necessary for the translation of genes that contain the rare codon TTA encoding for leucine. Therefore, it will affect only genes containing the TTA codon [39]. Point mutations in RpoB are known to improve secondary metabolites production by increasing the affinity of the RNA polymerase promoter, while in RpsL, however, protein synthesis is induced in the stationary growth phase, therefore improving the production of compounds [71]. The overexpression of wild-type rpsL also showed the activation of compounds in *S. clavuligerus* [38].

Sequence analysis software antiSMASH includes in its seventh version a prediction tool of transcription factors binding sites (TFBS) with which it was possible to know which gene clusters had a binding site for the regulators used in this work. Table S4 shows the TFBS predicted with strong confidence. Only clusters shown in Table 4 had a predicted TFBS for the regulators used in this work and showed a modified production level. As observed in Table S5, many clusters in all strains contain a TTA codon. However, only a few detected a modification in their production levels when introducing pSETxkBMRRH (Table 4). Interestingly, the holomycin cluster predicted in CS014, CS131, CS147, and CS149 are predicted to possess a TTA codon, but we were not able to detect the compound in any of these strains. Moreover, CS065a has a gene cluster with 100% similarity to the maltophilin biosynthetic gene cluster and does possess a TTA codon, but maltophilin was not detected in strain, but in strain CS090a, which possesses the same gene cluster also with a TTA codon; maltophilin was detected under the same conditions. The same situation was found in CS113 and CS159, but in the case of CS159, the cluster also has a TFBS for AbrC3. However, undecylprodigiosin is overproduced when carrying pSETxkBMRRH (which contains bldA) but not with pSETxkDCABA (which contains abrC3). Regulators interact with each other, making it impossible to predict the effect that these regulators will have on a particular strain. For example, glnR expression, which controls nitrogen metabolism, and other genes from nitrogen metabolism are controlled by PhoP, which controls phosphate metabolism and they both interact with AfsR, AfsQ1 (response regulator), or DasR (chitin degradation) [21,22,23,24]. As proven by the results of this work, the same regulators have a different effect in the same biosynthetic cluster, which is most probably due to the different regulatory cascades that each strain possesses. Therefore, the prediction of the effect that these plasmids would have on a particular strain is limited.

## 4. Conclusions

In the era of genetic manipulation, where -omics approaches are constantly applied, there is a bottleneck in terms of the discovery of new compounds that can serve as therapeutic alternatives or new treatments. The use of integrative recombinant plasmids bearing global regulators or housekeeping genes is a relatively simple strategy that allows the improvement of bioactive compound production which can also serve as a strategy for the activation of silenced metabolic pathways. It has been verified at an extensive experimental level that, by introducing pSETxkDCABA and pSETxkBMRRH into different wild-type strains from the CS collection and combining these constructs with ribosomal engineering, the biosynthesis of metabolites is enhanced or activated. Depending on the different sources of nutrients, the improvement in the production of compounds is more noticeable. Likewise, the combination of this strategy with others such as ribosomal engineering is proposed as an alternative to incorporate other technologies. It is important to take into account that, although there has been an increase in the production of metabolites at a general level, its increase is dependent on the strain, the culture, and extraction conditions, so the use of these constructs requires performing a screening of media and culture conditions to optimize the production of the compound of interest. Even though it is possible to predict binding sites for the regulators used in this work, as the results showed, it is not guaranteed that a specific cluster is going to be affected by the insertion of these plasmids due to the different regulation levels that each strain possesses.

## Figures and Tables

**Table 1:** Global regulators and their implication on *Streptomyces* regulation. ACT (actinorhodin), RED (undecylprodigiosin), CDA (calcium-dependent antibiotic), and yCPK (yellow-pigmented secondary metabolite). ↓↓ Indicates a decrease in production and ↑↑ indicates an increase in production.

Gene Product	Influence in *Streptomyces* Secondary Metabolism	Effect of Deletion in *S. coelicolor*	Effect of Overexpression in *S. coelicolor*	References
***CRP*** (*Cyclic AMP receptor protein*)	Regulation of the synthesis of several antibiotics	↓↓ ACT and CDA	↑↑ ACT, CDA and RED	[25]
***AfsR***	Pleiotropic regulatory protein for antibiotic production	↓↓ ACT and RED	Increase metabolite production ↑↑ ACT and RED	[26,27,28]
***BldD***	Control of morphological development and antibiotic production	↓↓ ACT, RED, and CDA. Deficiency in sporulation and antibiotic production	Not found	[29,30]
***DraR***	Activation/inhibition of antibiotic production	↑↑ yCPK, ↓↓ ACT	Not found	[26,31]
***AbrC3***	Activation of antibiotic production	↓↓ ACT and RED	↑↑ ACT	[32]

**Table 2:** Housekeeping genes and their effects on the metabolism of *Streptomyces* spp.

Gene	Gene Product	Effect on Antibiotic Production in *Streptomyces* spp.	References
***metK***	S-adenosylmethionine synthetase	Overproduction of ACT in *S. coelicolor* and streptomycin in *S. griseus* and 2-to-5-fold higher production of avermictin in *S. avermitillis.*	[33,34]
***hrdB***	RNA polymerase main sigma factor. Binds promoters of different secondary metabolism clusters	Induced expression of the gene showed increased promoter activity in *S. coelicolor*	[35,36]
***rpoB***	RNA polymerase β subunit	A point mutation in the gene activates antibiotic production in *S. lividans.* No information was found about its overexpression.	[37]
***rpsL***	Ribosomal protein S12	Point mutation in the gene resulted in an overproduction of antibiotics in *S. coelicolor*. Overexpression of wild-type *rpsL* also showed the activation of compounds in *S. clavuligerus*	[37,38]
***bdlA***	tRNA translates UUA codon to leucine	Overexpression of the gene in *S. coelicolor* activates the expression of silent clusters	[39]

**Table 3:** Metabolites identified from each strain used in this work and the effect that the insertion of the described vectors had in the metabolic production of each strain. In the case that the effect was not observed in all media and extraction methods, the growth and extraction conditions where the effect was detected are specified. (↑: overproduction; ↓: inhibition; (A) activation).

Strain	Known Compounds Identified by Dereplication	Insertion of pSETxkDCABA	Insertion of pSETxkBMRRH
*Streptomyces* sp. CS014	Granaticin A, granaticin C, collismycin C, collismycin B, collismycin A, collismycin D, cyclo (Tyr-Pro), N-acetyltiramine, cyclo (Leu-pro), pyrosulfoxin A, cyclo (Phe-Pro), alloesaponarin II, coproporphyrins	↑ Granaticins, collismycins	↑ Granaticins, collismycins
*Streptomyces* sp. CS057	Cycloheximide, actiphenol, skyllamycin A, skyllamycin B, coproporphyrins	↑ Skyllamycins, actiphenol, coproporphyrins, cycloheximide	↑ Skyllamycins, actiphenol, coproporphyrins, cycloheximide
*Streptomyces* sp. CS065a	Alteramides, chromomycin A3, chromomycin Ap, chromomycin A2, coproporphyrins	↓ Chromomycins only in SM10 media ↑ chromomycins in R5A, SM17 and SM20 (A) chromomycins in YEME-S	↑ Alteramides coproporphyrines in SM20
*Streptomyces* sp. CS081a	Dihydrotetrodecamycin, cosmomycin C, coproporphyrins	↑ Dihydrotetrodecamycin	↑ Cosmomycins (SM10 and SM17 extracted with ethyl acetate and ethyl acetate 1% formic acid)
*Streptomyces* sp. CS090a	Maltophilin, alteramides, 2-aminobenzoic acid, coproporphyrins	↑ Maltophilins (YEME-S), 2-aminobenzoic acid (SM10)	↑ Maltophilins (YEME-S and R5A), 2-aminobenzoic acid (SM10) (A) alteramides
*Streptomyces* sp. CS113	2,4-dihydro-2-hydroxy-1(2h)-isoquinolinone, germicidin A, cervimycin A, coproporphyrins, seitomycin, cyclo(phenylalanylprolyl), papuline, aurantimycin	-	↑ Germicidin (R5A and SM10
*Streptomyces* sp. CS131	Actinomycin D, I, G4, X2, coproporphyrins	↑ Actinomycins (m10, SM17, and R5A)	-
*Streptomyces* sp. CS147	Colibrimycins, vicenistatin, cyclo (leu-Pro), coproporphyrins, N-acetyltyramine, N-chloroacetyl tryptophan	↑ Antibiotic cyclo (leu-pro), colibrimycins, N-acetyltyramine ↑ coproporphyrins (SM17 and SM10) ↑ N-chloroacetyl tryptophan(R5A)	↑ Antibiotic cyclo (leu-pro), colibrimycins, N-acetyltyramine ↑ coproporphyrins (SM17 and SM10) ↑ N-chloroacetyl tryptophan(R5A)
*Streptomyces* sp. CS149	Collismycin A, B, C, D, F, coproporphyrins	Coproporphyrins (SM17 and SM10) ↑ bioactive compound against Gram-positive in R5A	Collismycins coproporphyrins ↑↑ bioactive compound against Gram-positive in R5A
*Streptomyces* sp. CS159	Undecylprodiogiosin, inthomycins, coproporphyrins	↑ Inthomycin (R5A)	↑ Inthomycin (R5A and SM10) ↑ undecylprodigiosin (R5A)
*Streptomyces* sp. CS207	3-(2-hydroxiethyl)-6-prenylindole, 3-cyanomethyl-6-prenylindole, coproporphyrins	↑ 3-cyanomethyl-6-prenylindone, 3-(2-hydroxyethyl)-6-prenylindole (R5A, ethyl acetate, ethyl acetate 1% formic acid) ↑ coproporphyrins (SM17 and R5A)	↑ 3-cyanomethyl-6-prenylindone, 3-(2-Hydroxyethyl)-6-prenylindole (R5A, ethyl acetate, ethyl acetate 1% formic acid)
*Streptomyces* sp. CS227	Surugamide A, 2-aminobenzoic acid, abenquine A, coproporphyrins	↑ 2-aminobenzoic acid and surugamide A	↑ 2- aminobenzoic acid and surugamide A

**Table 4:** Clusters in each strain that have a predicted TFBS and had their product levels modified when inserting pSETxkDCABA and/or pSETxkBMRRH.

Strain	Gene Cluster Number	Gene Cluster Type and MiBiG Identification	TFBS
CS014	1.6	NRPS/PKS-I (Collismycin A 77%)	*bldA*
CS057	1.2	NRPS-like/lanthipeptide- IV/transAT-PKS (Cycloheximide 94%)	*bldA*
	1.20	Ectoine/butyrolactone/ladderane/arylpolyene/NRPS/PKS-I (Skyllamycins 97%)	*bldA*
CS065	1.23	PKS-II/oligosaccharide (Chromomycin 100%)	*bldA*
CS081a	1.4	PKS-II/oligosaccharide/PKS-like (Cosmomycin D 97%)	*bldA*
CS090a	1.21	NRPS/PKS-I (Maltophilin 100%)	*bldA*
CS113	1.22	PKS-II/PKS-like/oligosaccharide (Cervimycin 90%)	AbrC3
	1.26	Butyrolactone/PKS-III (Germicidin 100%)	AfsR
			BldD
			AbrC3
			*bldA*
CS131	1.6	NRPS-like/other (Actinomycin 89%)	AbrC3
			*bldA*
CS147	1.16	Ladderane/NRPS (Colibrimycin 75%)	*bldA*
	1.17	PKS-I/RRE-element containing cluster (Vicenstatin 100%)	*bldA*
CS149	1.21	NRPS/PKS-I (Collismycin A 70%)	*bldA*
CS159	1.5	Trans-AT PKS/NRPS/NRPS-like (Inthomycin B 100%)	*bldA*
	1.10	NRP-metallophore/NRPS/PKS-I/NRPS-like/prodigiosin (Undecylprodigiosin 100%)	*bldA*

## Data Availability

Data is contained within the article or supplementary material.

## Supplementary Materials

The following supporting information can be downloaded at: https://www.mdpi.com/article/10.3390/microorganisms11061585/s1, Table S1: Streptomyces strains used in this study; Table S2: Primers used in this study. Table S3: Primers used for strain confirmation. Figure S1: Comparative UPLC analysis of CS065a samples Figure S2: Comparative UPLC analysis of CS081a samples. Figure S3: Comparative UPLC analysis of CS090a samples. Figure S4: Comparative UPLC analysis of CS131 samples. Figure S5: Comparative UPLC analysis of CS159 samples. Figure S6: Comparative UPLC analysis of C207 samples. Figure S7: Comparative UPLC analysis of C227 samples. LC-MS dereplication: Data corresponding to the identification of compounds whose production was activated in this work. Table S4: Transcription Factor Binding Sites (TFBS) involved in this work find with strong confidence in the chromosome of each strain using antiSmash 7.0. Table S5: TTA codons find in the chromosome of each strain using antiSmash 7. References [72,73,74,75,76,77,78,79,80] are cited in the supplementary materials.
