# Supplementary material for: Co-Expression of Transcriptional Regulators and Housekeeping Genes in Streptomyces spp.: A Strategy to Optimize Metabolite Production
Source: Microorganisms. 2023 Jun 15;11(6):1585. doi: 10.3390/microorganisms11061585 (PMC10301460; doi:10.3390/microorganisms11061585)
Supplement: Supplementary file 1 [file microorganisms-11-01585-s001.zip › microorganisms-2396904-supplementary.pdf]

Table S1: *Streptomyces* strains used in this study

Strain	Construct	Reference
Streptomyces sp. CS014	WT	[14,15,57]
Streptomyces sp. CS057	WT	[14,15,57]
Streptomyces sp. CS065a	WT	[14,15,57]
Streptomyces sp. CS081a	WT	[14,15,57]
Streptomyces sp. CS090a	WT	[14,15,57]
Streptomyces sp. CS113	WT	[14,15,57]
Streptomyces sp. CS131	WT	[14,15,57]
Streptomyces sp. CS147	WT	[14,15,57]
Streptomyces sp. CS149	WT	[14,15,57]
Streptomyces sp. CS159	WT	[14,15,57]
Streptomyces sp. CS207	WT	[14,15,57]
Streptomyces sp. CS227	WT	[14,15,57]
Streptomyces sp. CS014 pSETxkDCABA	pSETxkDCABA: *draR, CRP, abrC3, bldD, afsR*	This work
Streptomyces sp. CS057 pSETxkDCABA	pSETxkDCABA: *draR, CRP, abrC3, bldD, afsR*	This work
Streptomyces sp. CS065a pSETxkDCABA	pSETxkDCABA: *draR, CRP, abrC3, bldD, afsR*	This work
Streptomyces sp. CS081a pSETxkDCABA	pSETxkDCABA: *draR, CRP, abrC3, bldD, afsR*	This work
Streptomyces sp. CS090a pSETxkDCABA	pSETxkDCABA: *draR, CRP, abrC3, bldD, afsR*	This work
Streptomyces sp. CS113 pSETxkDCABA	pSETxkDCABA: *draR, CRP, abrC3, bldD, afsR*	This work
Streptomyces sp. CS131 pSETxkDCABA	pSETxkDCABA: *draR, CRP, abrC3, bldD, afsR*	This work
Streptomyces sp. CS147 pSETxkDCABA	pSETxkDCABA: *draR, CRP, abrC3, bldD, afsR*	This work
Streptomyces sp. CS149 pSETxkDCABA	pSETxkDCABA: *draR, CRP, abrC3, bldD, afsR*	This work
Streptomyces sp. CS159 pSETxkDCABA	pSETxkDCABA: *draR, CRP, abrC3, bldD, afsR*	This work

Streptomyces sp. CS207 pSETxkDCABA	pSETxkDCABA: *draR, CRP, abrC3, bldD, afsR*	This work
Streptomyces sp. CS227 pSETxkDCABA	pSETxkDCABA: *draR, CRP, abrC3, bldD, afsR*	This work
Streptomyces sp. CS014 pSETxkBMRRH	pSETxkBMRRH: *bldA, metK, rpsL, rpoB, hrdB*	This work
Streptomyces sp. CS057 pSETxkBMRRH	pSETxkBMRRH: *bldA, metK, rpsL, rpoB, hrdB*	This work
Streptomyces sp. CS065a pSETxkBMRRH	pSETxkBMRRH: *bldA, metK, rpsL, rpoB, hrdB*	This work
Streptomyces sp. CS081a pSETxkBMRRH	pSETxkBMRRH: *bldA, metK, rpsL, rpoB, hrdB*	This work
Streptomyces sp. CS090a pSETxkBMRRH	pSETxkBMRRH: *bldA, metK, rpsL, rpoB, hrdB*	This work
Streptomyces sp. CS113 pSETxkBMRRH	pSETxkBMRRH: *bldA, metK, rpsL, rpoB, hrdB*	This work
Streptomyces sp. CS131 pSETxkBMRRH	pSETxkBMRRH: *bldA, metK, rpsL, rpoB, hrdB*	This work
Streptomyces sp. CS147 pSETxkBMRRH	pSETxkBMRRH: *bldA, metK, rpsL, rpoB, hrdB*	This work
Streptomyces sp. CS149 pSETxkBMRRH	pSETxkBMRRH: *bldA, metK, rpsL, rpoB, hrdB*	This work
Streptomyces sp. CS159 pSETxkBMRRH	pSETxkBMRRH: *bldA, metK, rpsL, rpoB, hrdB*	This work
Streptomyces sp. CS147 pSETxkBMRRH	pSETxkBMRRH: *bldA, metK, rpsL, rpoB, hrdB*	This work
Streptomyces sp. CS149 pSETxkBMRRH	pSETxkBMRRH: *bldA, metK, rpsL, rpoB, hrdB*	This work
Streptomyces sp. CS159 pSETxkBMRRH	pSETxkBMRRH: *bldA, metK, rpsL, rpoB, hrdB*	This work
Streptomyces sp. CS207 pSETxkBMRRH	pSETxkBMRRH: *bldA, metK, rpsL, rpoB, hrdB*	This work
Streptomyces sp. CS227 pSETxkBMRRH	pSETxkBMRRH: *bldA, metK, rpsL, rpoB, hrdB*	This work
Streptomyces sp. CS14 streptomycin resistant (S.R.)	WT	This work
Streptomyces sp. CS14 streptomycin resistant (S.R.) pSETxkDCABA	pSETxkDCABA: *draR, CRP, abrC3, bldD, afsR*	This work
Streptomyces sp. CS14 streptomycin resistant (S.R.) pSETxkBMRRH	pSETxkBMRRH: *bldA, metK, rpsL, rpoB, hrdB*	This work
Streptomyces sp. CS065a rifampicin resistant (R.R.)	WT	This work

Streptomyces sp. CS065 rifampicin resistant (R.R.) pSETxkDCABA	pSETxkDCABA: *draR, CRP, abrC3, bldD, afsR*	This work
Streptomyces sp. CS065a rifampicin resistant (R.R.) pSETxkBMRRH	pSETxkBMRRH: *bldA, metK, rpsL, rpoB, hrdB*	This work
Streptomyces sp. CS065a streptomycin resistant (S.R.)	WT	This work
Streptomyces sp. CS065a streptomycin resistant (S.R.) pSETxkDCABA	pSETxkDCABA: *draR, CRP, abrC3, bldD, afsR*	This work
Streptomyces sp. CS065a streptomycin resistant (S.R.) pSETxkBMRRH	pSETxkBMRRH: *bldA, metK, rpsL, rpoB, hrdB*	This work
Streptomyces sp. CS147 rifampicin resistant (R.R.)	WT	This work
Streptomyces sp. CS147 rifampicin resistant (R.R.) pSETxkDCABA	pSETxkDCABA. *draR, CRP, abrC3, bldD, afsR*	This work
Streptomyces sp. CS147 rifampicin resistant (R.R.) pSETxkBMRRH	pSETxkBMRRH. *bldA, metK, rpsL, rpoB, hrdB*	This work

Table S2: Primers for the generation of the plasmids used in this study.

**Name**	**Sequence 5-->3**
d-KAS-check	GTGTTGTAAAGTCGTGGCCAGG
rvKAS-check	TGTGGAATTGTGAGCGGATA
SmaI-NsiI-REP	ATATCCCGGGATGCATTGAGTTGAAGAGGTGACGTCA
MunI-REP	ATATCAATTGACGAATTCGAGCTCGGTACC
BglII-KasOd	GTGTAGATCTTGTAAAACGACGGCCAGTG
AbrC3 Fw Ndei	AATACATATGACCTGTCGCACGGACGGTTC
AbrC3 Rv ecorv bcui	AATGATATCATACTAGTGGTGCTTCCGAGGCATGG
AfsR Fw BcuI	ATATACTAGTCTGACGTGGTTGCTCAGGATG
AfsR Rv AanI	ATATTTATAATCACCGCGCCACACTGC
BldA Fw BamHI	ATATGGATCCTGGAACCTTCACCGATGGT
BldA Rv NotI	ATATGCGGCCGCGCCCGGATGGTGGAATG
BldD Fw BcuI AanI	AATTACTAGTATTTATAACCGCGTCGACACCTTGTCC
BldD Rv EcoRV	AATTGATATCTCAGAGCTCGTCGTGGGA
CRP-FW NotI	AATTGCGGCCGCAGCCGGTCGACAAGGAGAG
CRP-Rv NsiI	AATTATGCATTCAGCGGGAGCGCTTGG
DraR Fw BamHI	ATATGGATCCCGATCTTGCCCCGGGCGTTG
DraR Rv XK NotI	AATTGCGGCCGCCTCTTACCCGGAAGGCCCTC
HrdB Fw EcorV	ATATGATATCGCCGGAAGCCGTTTTTCAAC
HrdB Rv BcuI	ATATACTAGTGTACCGCCGGTCCGTACG

MetK Fw NotI	ATATGCGGCCGCCGTCCACCAATGATCAGCCA
Metk Rv NsiI	ATATATGCATGTGCGCCCCGGATCCTTA
RpoB Fw NdeI	AATACATATGGCGCGCGTAGTGAGTCCG
RpoB Rv EcoRV BcuI	ATATGATATCATACTAGTTGACTCCCGTCAGACCTCTT
RpsL Fw NsiI	AATTATGCATCTTCACCATTCGGCACACAGAAACC
RpsL Rv NdeI	AATACATATGTTACTTCTCCTTCTTGGCGCCGTA

Table S3: Primers used for strain confirmation. DraR Fw and Crp Rv, afsR Fw and bldD Rv and bldD Fw and Neo Rv were used to confirm the presence of pSETXK-DCABA and bldA Fw and metK Rv, rpoB Rv and rpsL Fw and rpoB Fw and hrdB Rv were used to confirm the presence of pSET BMRRH.

Construct checked	Genes amplified	Primers	Sequence
**pSETxkDCABA**	*draR, CRP*	**draR Fw**<br>**Crp Rw**	ATATGGATCCCGATCTTGCCCCGGGCGTTG<br>AATTATGCATTCAGCGGGAGCGCTTGG
	afsR, bldD	**afsR Fw**<br>**bldD Rw**	GAGCACCTTGCCGTTGTAGT<br>GACACCTCGCTGCTCGAA
	bldD, abRC3	**bldD Fw**<br>**Neo Rw**	AATTACTAGTATTTATAACCGCGTCGACACCTTGTCC<br>ATTAAGAGCTCGAACCCCAGAGTCCCGCTCAG
**pSETxkBMRRH**	*bldA, metK*	**bldA Fw**<br>**metK Rw**	ATATGGATCCTGGAACCTTCACCGATGGT<br>ATATGCGGCCGCCGTCCACCAATGATCAGCCA
	rpsL, rpoB	**rpsL Fw**<br>**rpoB Rw**	GCAGGACAAGGTCGAGAAGA<br>CTGGTGCCGAAGAACTCCT

	rpoB, hrdB	**rpoB Fw**<br>**hrdB Rw**	GCAGGACAAGGTCGAGAAGA<br>GCAGGACAAGGTCGAGAAGA

Additional results obtained

CS065a

An overproduction of chromomycins was detected in CS065a, both strains in different media, except for the strain containing pSETxkDCABA grown in SM10 where chromomycin production was inhibited, which will be discussed in the Inhibition section. Chromomycins have anti-cancer activity and are widely used as DNA staining since they bind to GC rich DNA [1]. In the culture of strain bearing pSETxkBMRRH grown in SM20 media, the antifungal compounds alteramides [2], and coproporphyrins were also overproduced (Figure S1, Supplementary material).

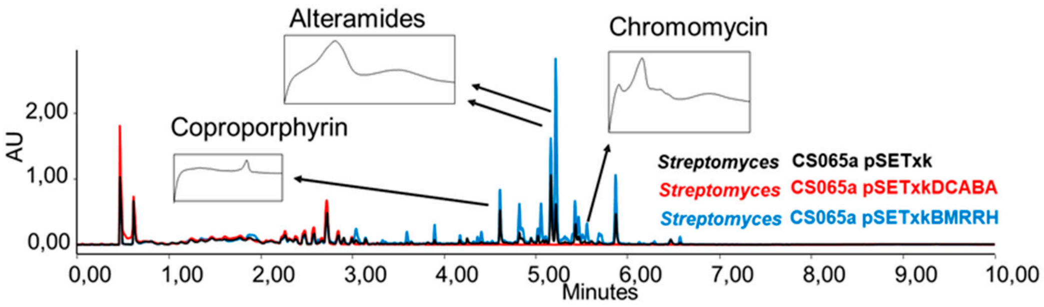

Figure S1. Comparative UPLC analysis of CS065a samples cultured in liquid SM20 medium at day 8 of culture and extracted with ethyl acetate: overproduction of chromomycin, alteramides, and coproporphyrin by pSETxkBMRRH construct can be observed. UV-Vis spectra of these compounds are shown.

CS081a:

CS081a: Cultivation of CS081a carrying pSETxkBMRRH construct in SM10 and SM17 media and extracted with ethyl acetate or acetyl acetate with 1% formic acid, showed an increased production of antitumor compound cosmomycin D [3]. Additionally, the recombinant strain bearing pSETxkDCABA also showed an increased production of dihydrotetrodecamycin (Figure S2, Supplementary material).

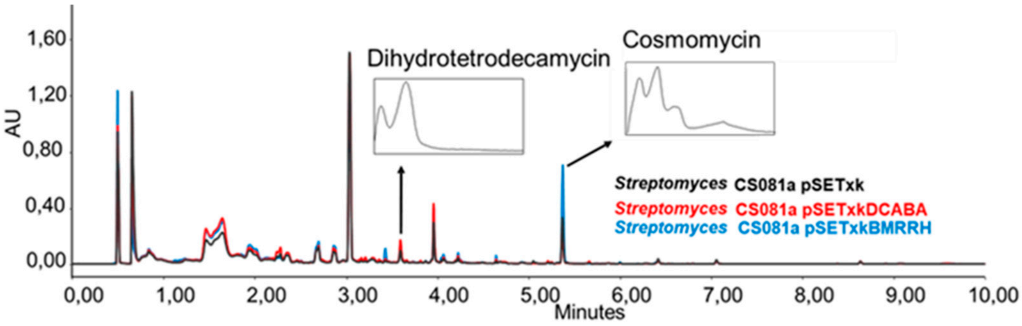

Figure S2 Comparative UPLC analysis of CS081a samples cultured in liquid SM10 medium on day 8 of culture and extracted with ethyl acetate with 1% formic acid: overproduction of cosmomycin by CS081a pSETxkBMRRH can be observed. Both recombinants overproduce dihydrotetrodecamycin. UV-Vis spectra of these compounds are shown.

CS090a:

CS090a: CS090a carrying pSETxkDCABA or pSETxkBMRRH cultured on agar YEME-S and extracted with any of the tested solvents showed an overproduction of maltophilins antibiotics [4] and alteramides. *Streptomyces* CS090a pSETxkBMRRH cultured on agar R5A (discussed in the Activation section) overproduced maltophilins and activated the biosynthesis of alteramides. Furthermore, when grown in SM10 media, both mutants overproduced the bioactive compound 2-aminobenzoic acid also known as anthranilic acid or vitamin L1 [5-7](Figure S3, Supplementary material).

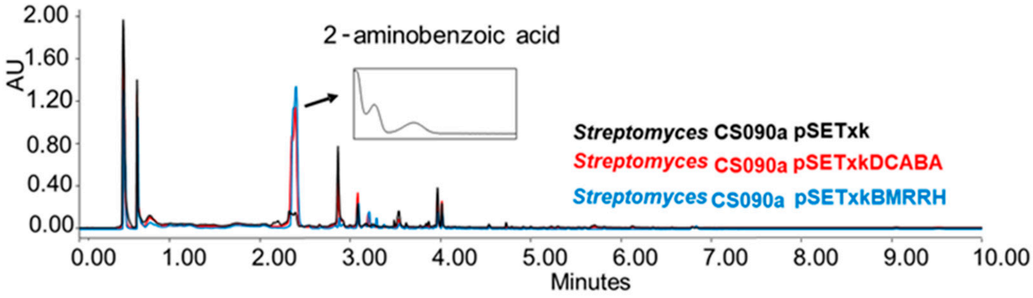

Figure S3. Comparative UPLC analysis of CS090a samples cultured in liquid SM10 medium at day 4 of cultivation and extracted with ethyl acetate with 1% formic acid: overproduction of 2-aminobenzoic acid by both mutants can be observed. UV-Vis spectrum of this compound is shown.

CS131:

CS131: An increased production of antibiotic actinomycin D was detected in recombinants carrying any of the constructs and grown in SM17, R5A, and SM10 media [8,9]. Furthermore, it was observed, especially in R5A, an activation of the production of different types of actinomycins (actinomycin I and actinomycin G4) that the wild strain does not produce or does it in undetectable levels under the conditions and with the methodology tested (Figure S4, Supplementary material).

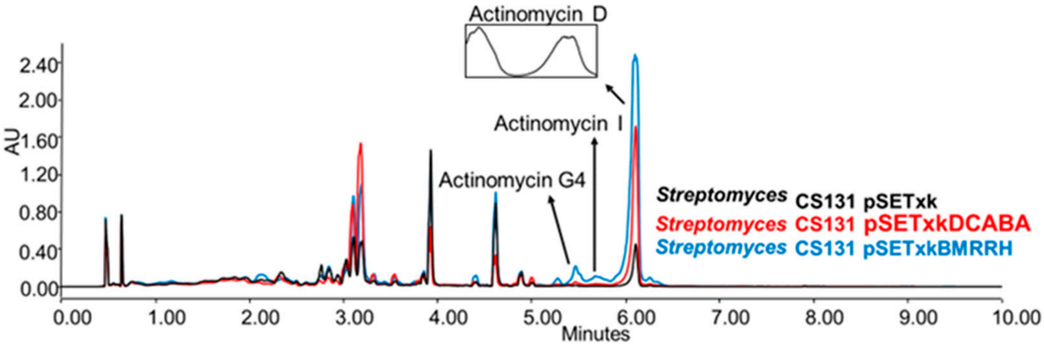

Figure S4: Comparative UPLC analysis of CS131 samples cultured in liquid R5A medium at day 13 of culture and extracted with ethyl acetate with 1% formic acid: overproduction of actinomycins by both mutants can be observed. UV-Vis spectrum of these compounds is shown.

CS159:

CS159: Both recombinants of this strain overproduced herbicide and antifungal compound inthomycin when cultured in R5A [10]. The strain carrying pSETxkBMRRH also overproduced this compound in SM10 and SM17. In addition, the overproduction of the antibiotic undecylprodigiosin can be observed in the R5A culture (Figure S5, Supplementary material) [11].

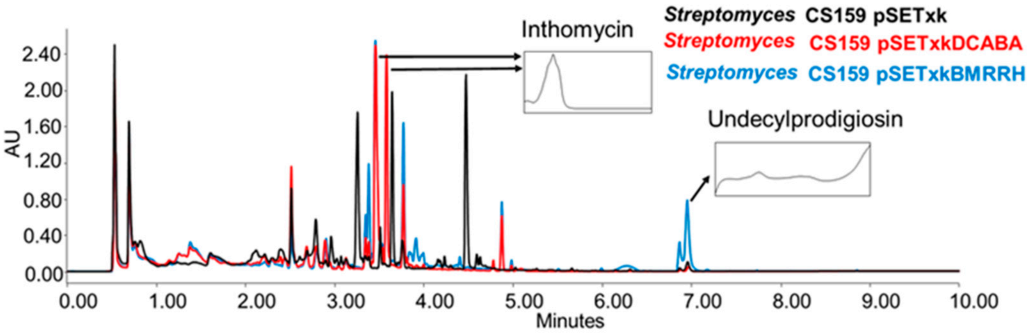

Figure S5. Comparative UPLC analysis of CS159 samples cultured in liquid R5A medium at day 13 of culture and extracted with ethyl acetate: overproduction of inthomycin by both clones and undecylprodigiosin by pSETxkBMRRH construct can be observed. UV-Vis spectra of these compounds are shown.

CS207:

CS207: Both recombinants of this strain overproduced derivative forms of bioactive compoun prenylindol, such as 3-cyanomethyl-6-prenylindone and 3-(2-Hydroxyethyl)-6-prenylindole when cultured in R5A(extracted with both ethyl acetate or ethyl acetate with 1% formic acid). In addition, in SM17 and R5A the pSETxkDCABA clone overproduces coproporphyrins (Figure S6, Supplementary material) [60].

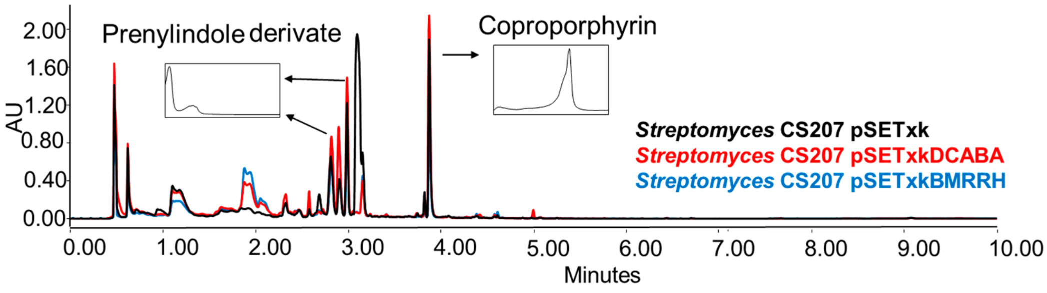

Figure S6. Comparative UPLC analysis of C207 samples cultured in liquid R5A medium and extracted with ethyl acetate with 1% formic acid: overproduction of several prenylindole derivatives by both clones could be observed. Moreover, increased production of coproporphyrins by pSETxkDCABA carrying strain can be observed. UV-Vis spectra of these compounds are shown.

CS227:

CS227: Overproduced 2- aminobenzoic acid and surugamide A in all the media tested by both recombinants (Figure S7, supplementary material)

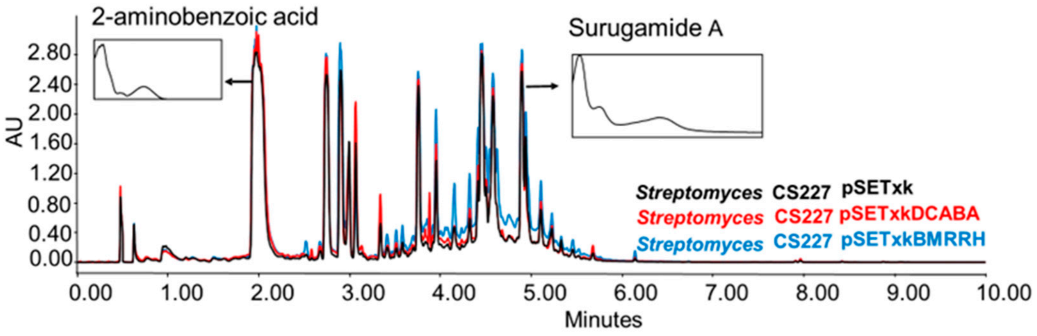

Figure S7. Comparative UPLC analysis of CS227 samples cultured in liquid SM17 medium and extracted with ethyl acetate: overproduction of 2-aminobezoic and surugamide by both strains can be observed. UV-Vis spectrums of these compounds are shown.

LC-MS dereplication: **Data corresponding to the identification of compounds whose production was activated in this work**

CS014:

Collismycin A-B, $C_{13}H_{13}N_3O_2S$:

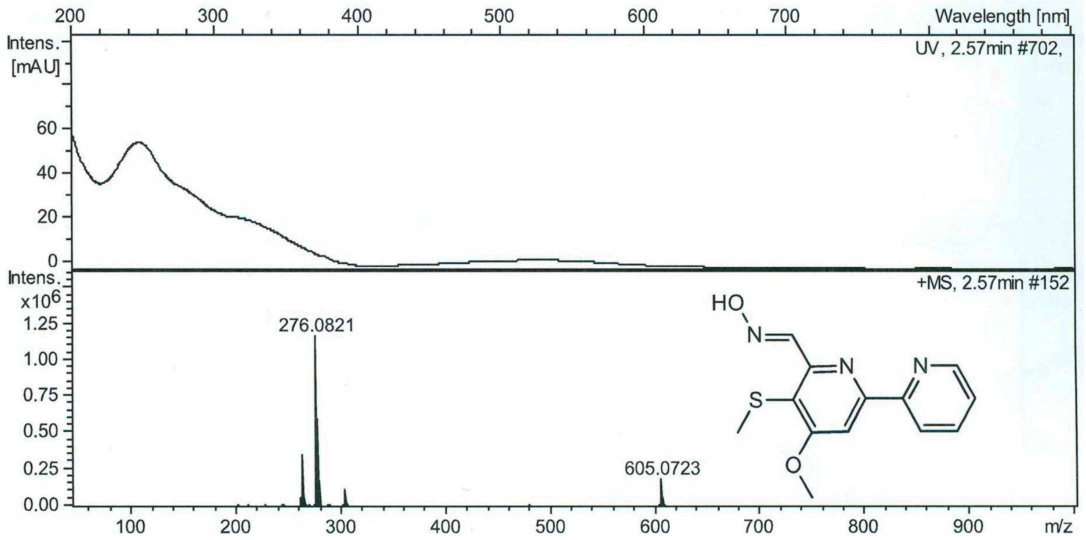

Collismycin C, $C_{13}H_{14}N_2O_2S$:

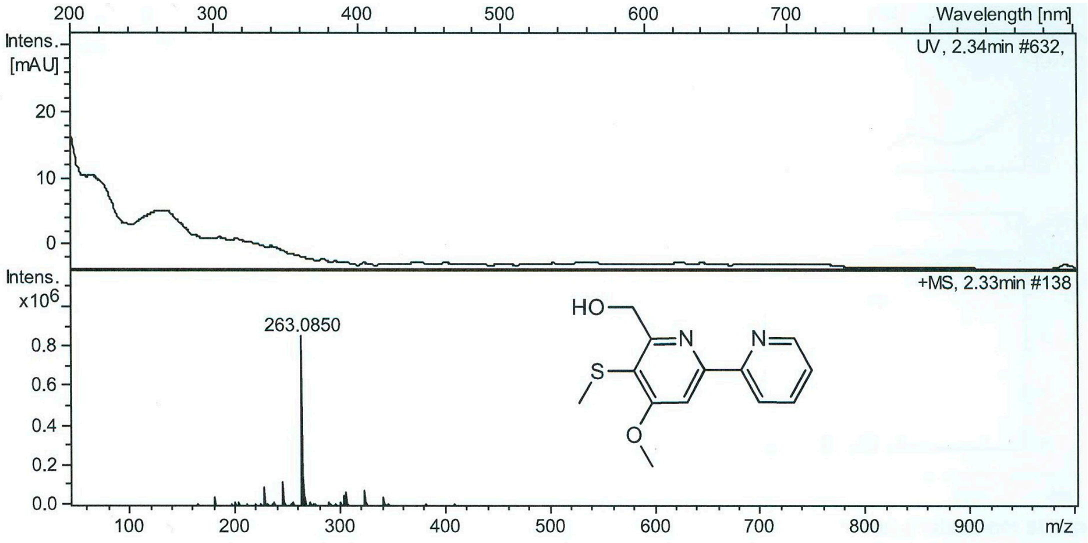

Granaticin A, $C_{22}H_{20}O_{10}$:

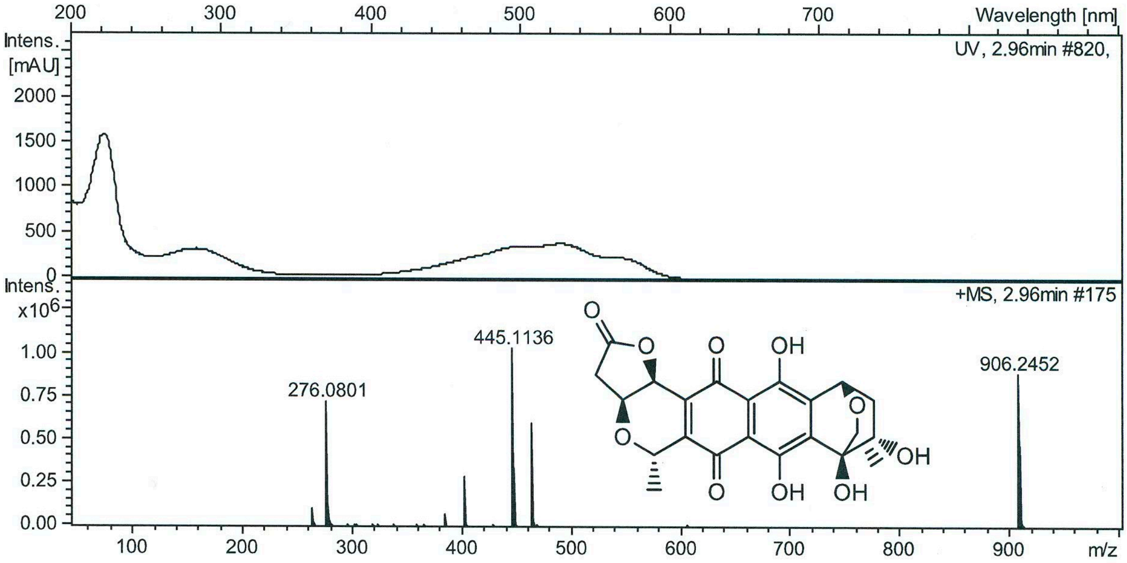

Granaticin C, $C_{28}H_{30}O_{12}$:

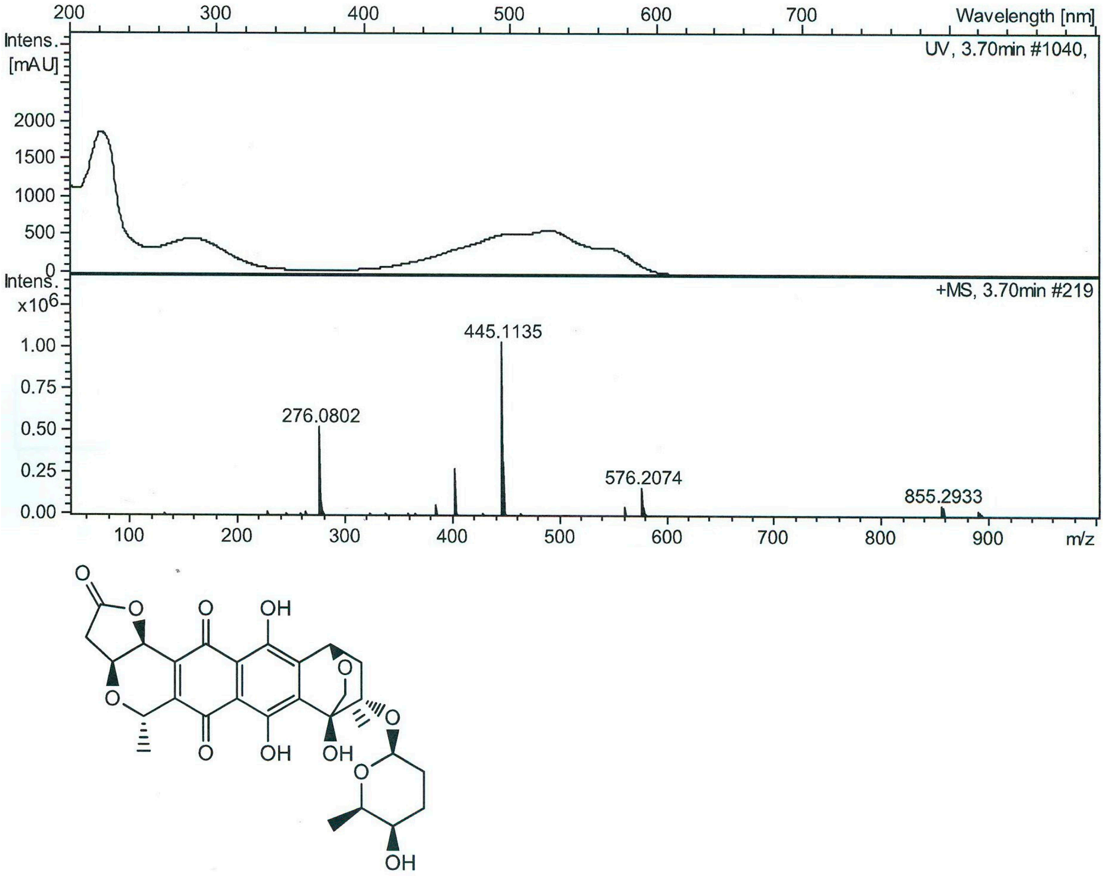

CS057:

Cycloheximide, $C_{15}H_{23}NO_4$:

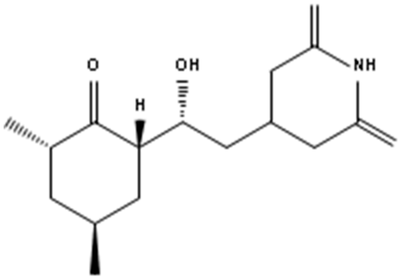

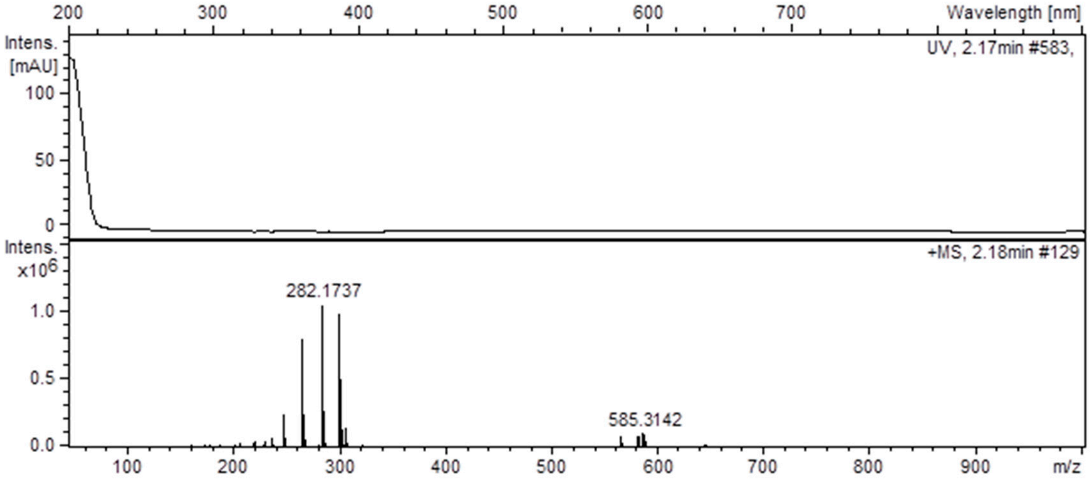

Actiphenol, $C_{15}H_{17}NO_4$:

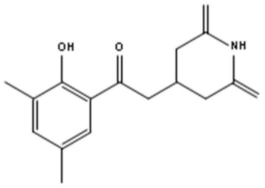

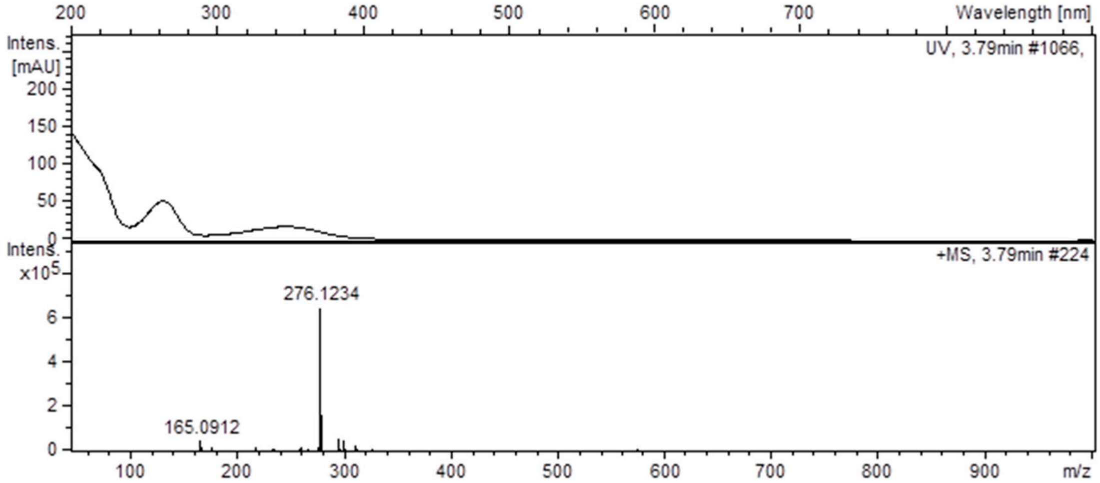

Skyllamycin A, $C_{75}H_{94}N_{12}O_{20}$

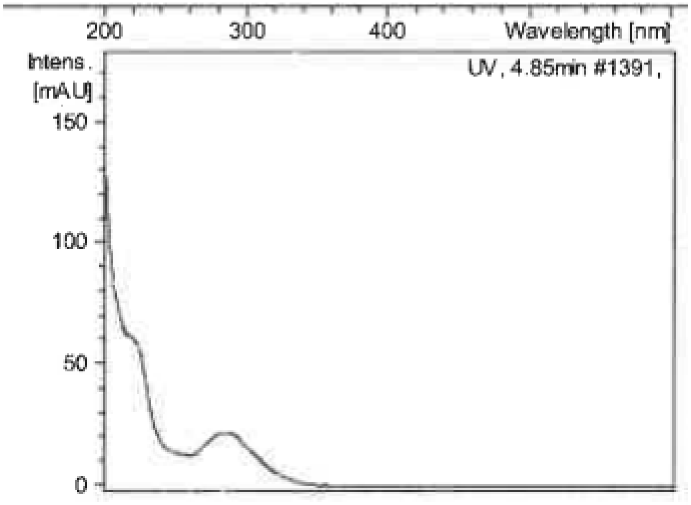

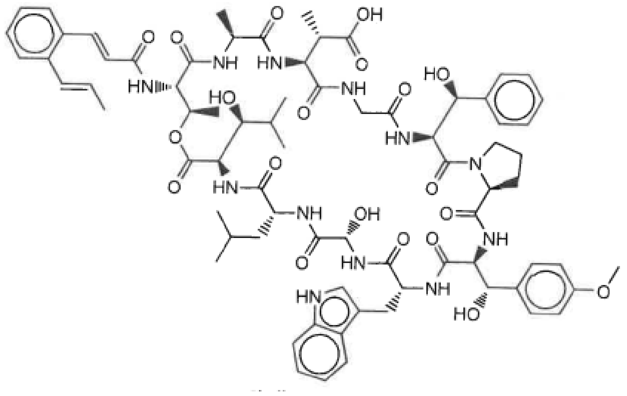

Skyllamycin B, $C_{74}H_{92}N_{12}O_{20}$

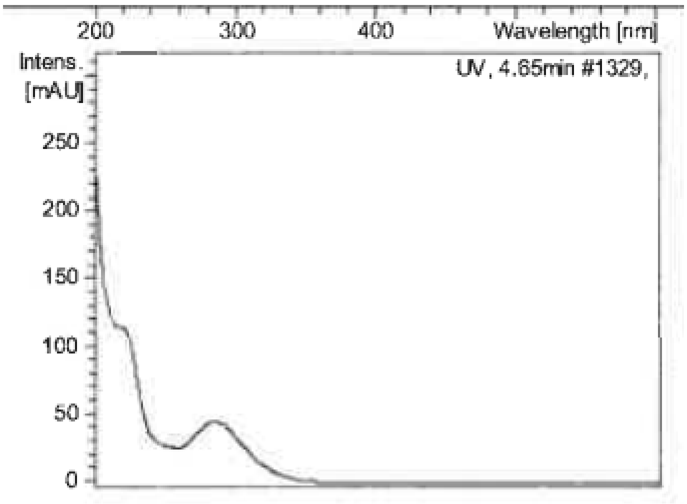

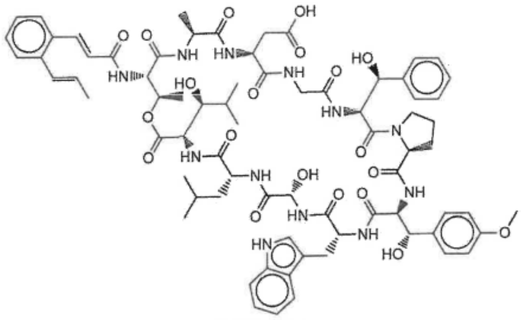

CS065a:

Alteramide A, $C_{29}H_{38}N_2O_6$:

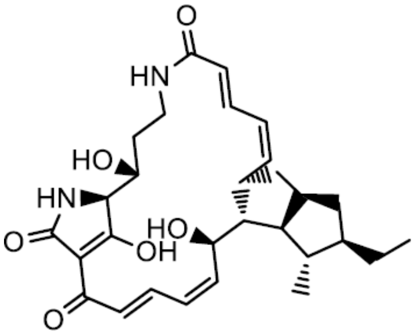

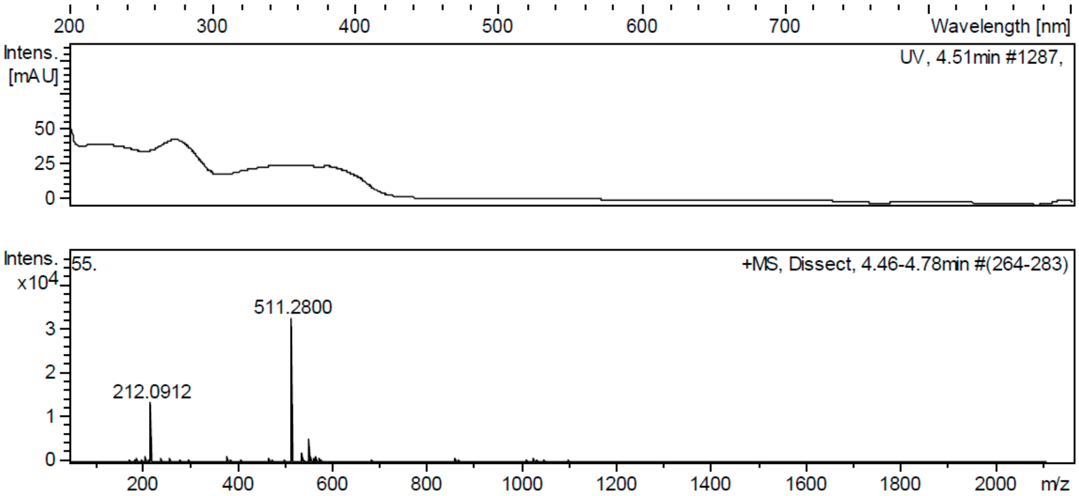

Chromomycin A3, $C_{57}H_{82}O_{26}$:

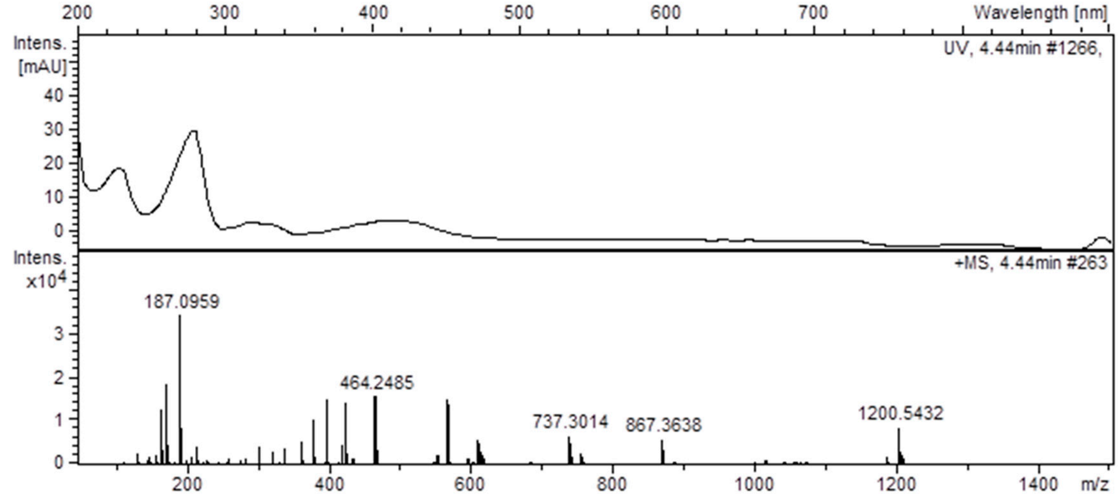

CS081a:

Dihydrotetrodecamycin, $C_{18}H_{24}O_{6}$:

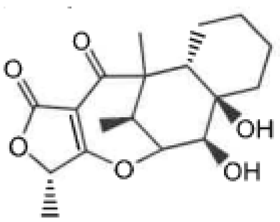

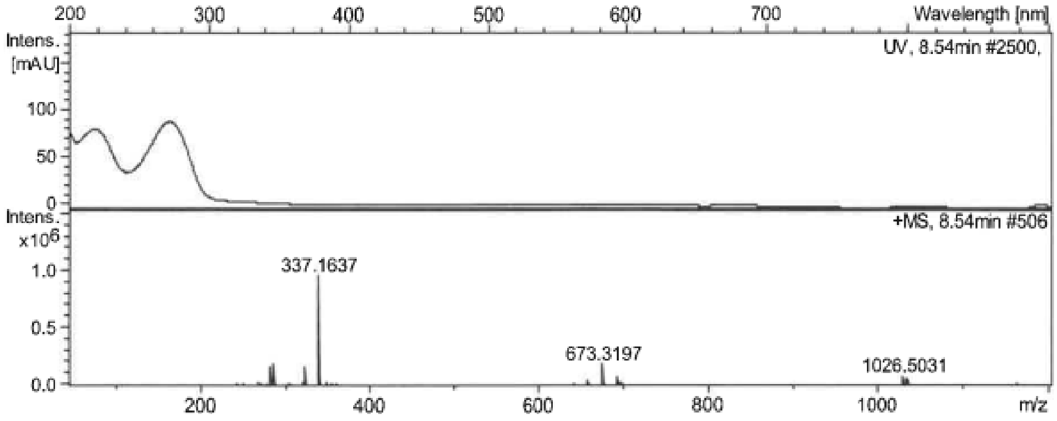

Cosmomycin D, $C_{60}H_{88}N_2O_{22}$:

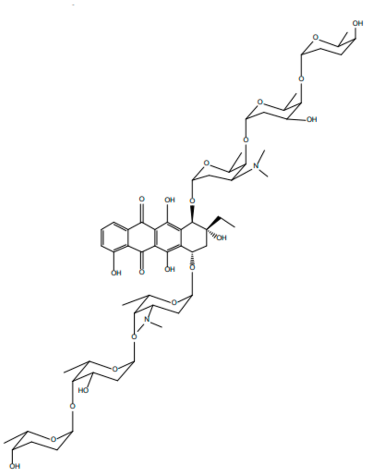

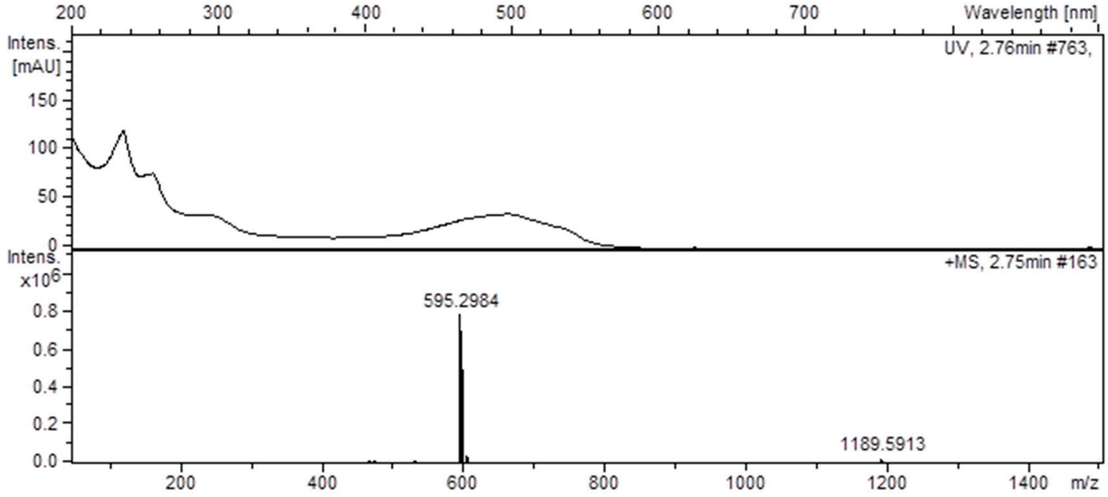

CS090a:

Maltophilin, $C_{29}H_{38}N_2O_6$:

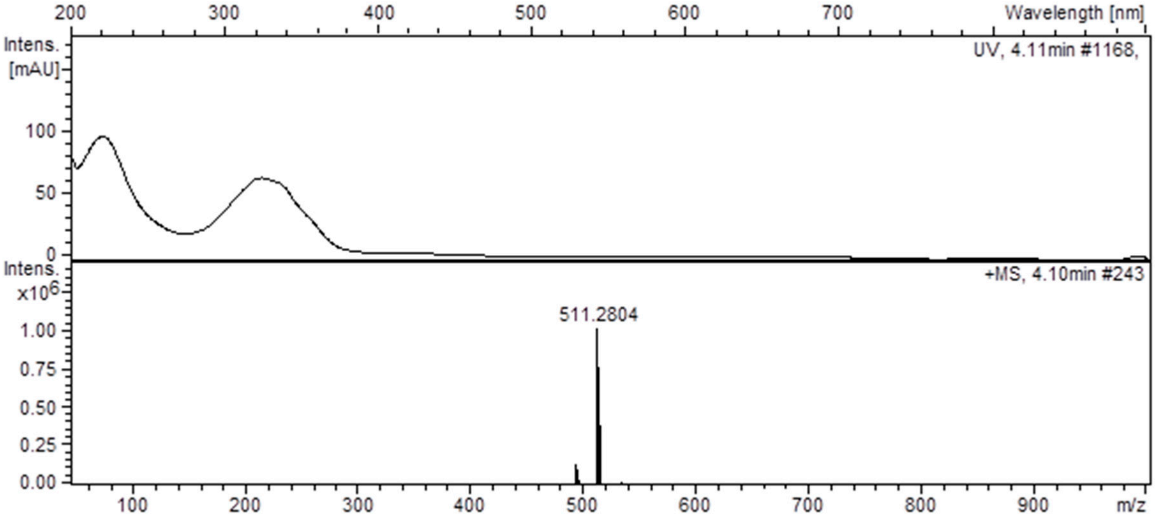

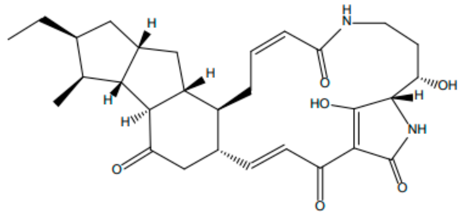

Alteramide A, $C_{29}H_{38}N_2O_6$:

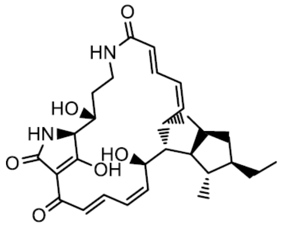

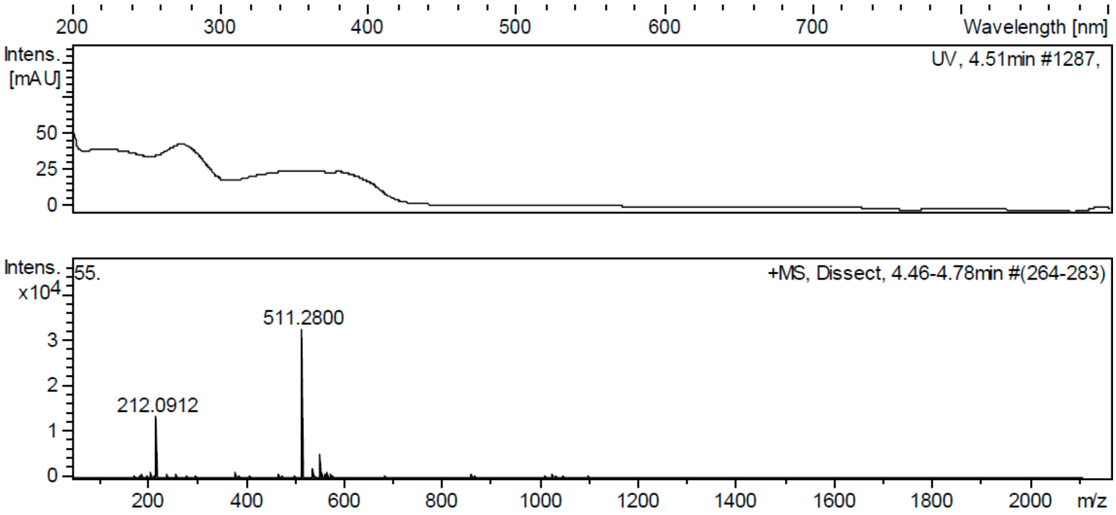

2-aminobenzoic acid, $C_7H_7NO_2$:

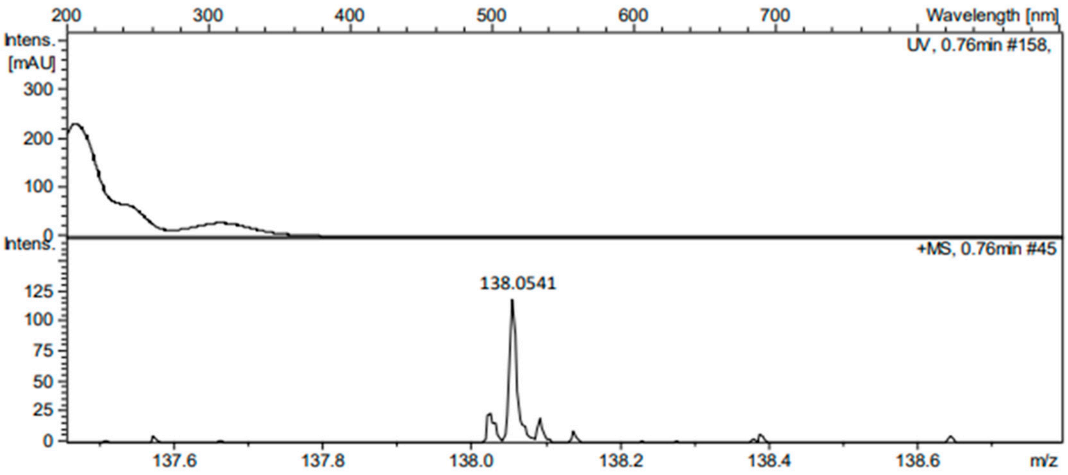

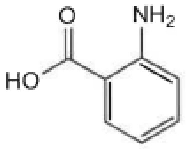

CS113:

Germicidin A, $C_{11}H_{16}O_3$:

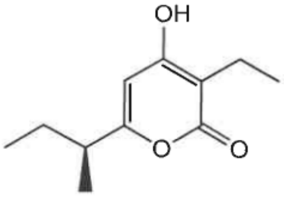

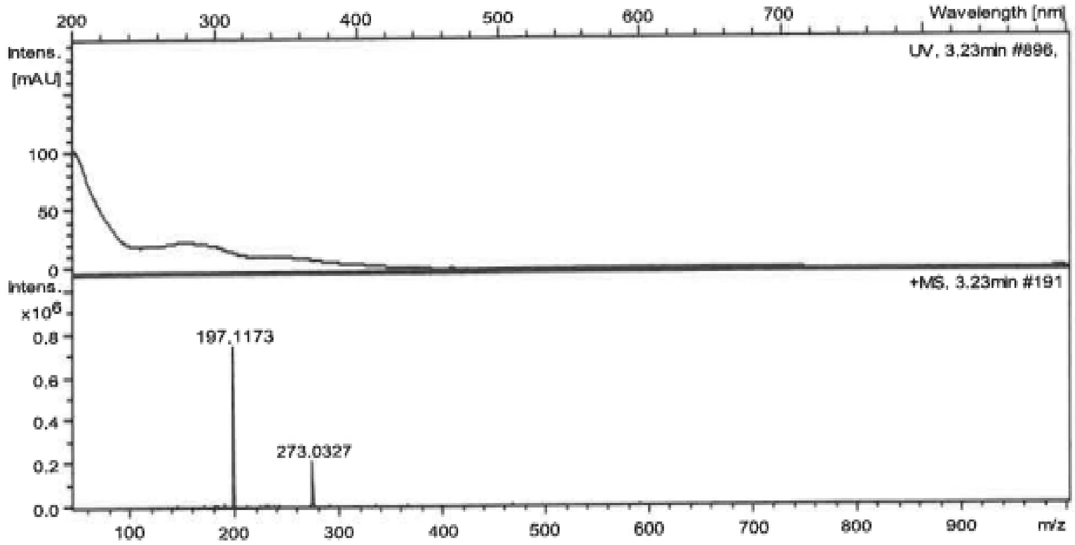

CS131:

Actinomycin D, $C_{62}H_{86}N_{12}O_{16}$:

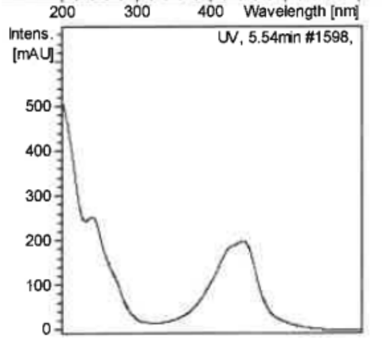

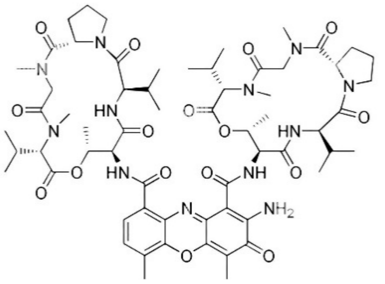

Actinomycin I, $C_{62}H_{86}N_{12}O_{17}$:

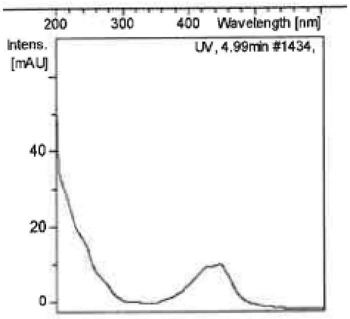

Actinomycin G4, $C_{61}H_{84}N_{12}O_{17}$:

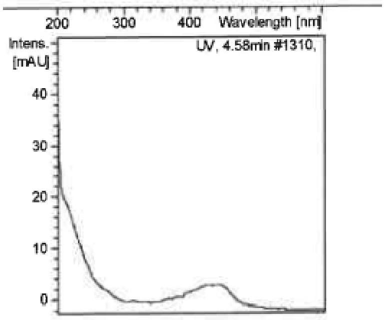

CS147:

N-Chloroacetyl tryptophan, $C_{13}H_{13}ClN_2O_3$:

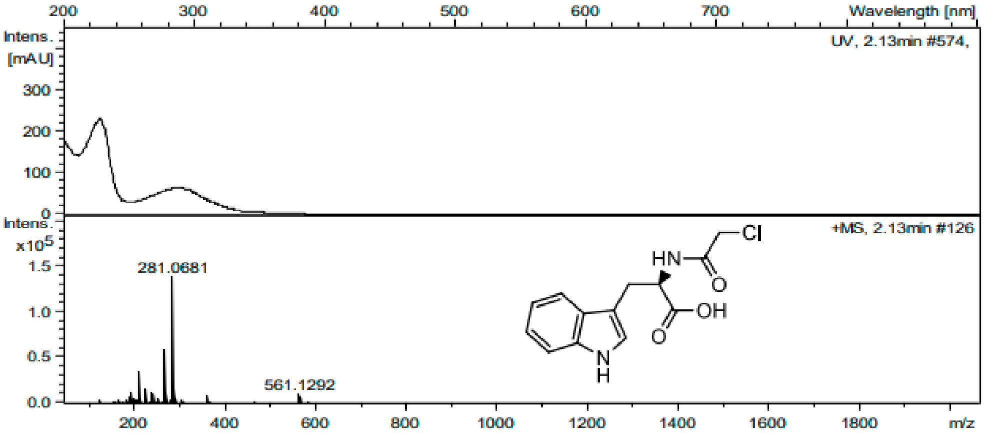

N-acetyltyramine, $C_{10}H_{13}NO_2$

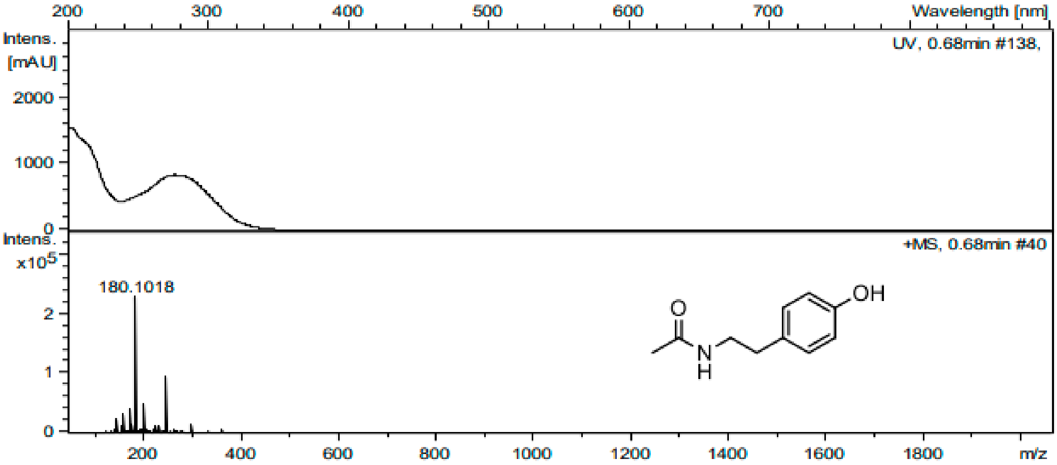

Cyclo (leu-pro), $C_{11}H_{18}N_2O_2$:

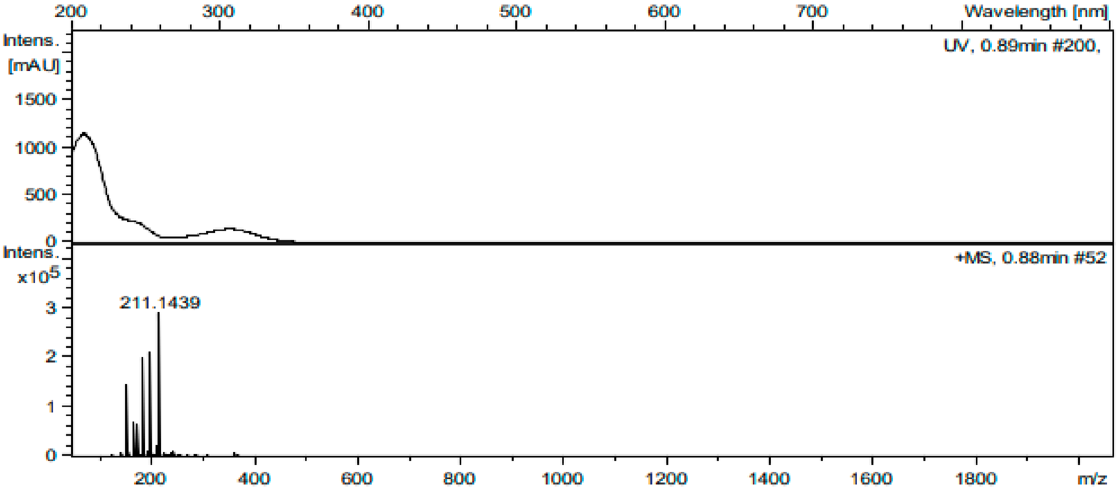

Vicenistatin, $C_{30}H_{48}N_2O_4$:

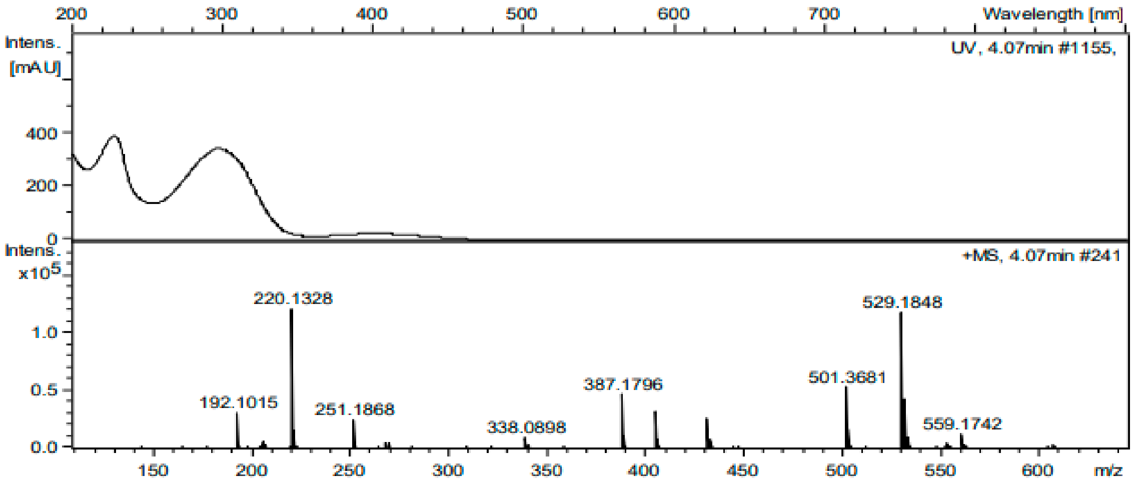

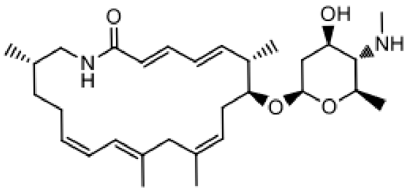

Colibrimycin A1, $C_{27}H_{30}N_5O_8$:

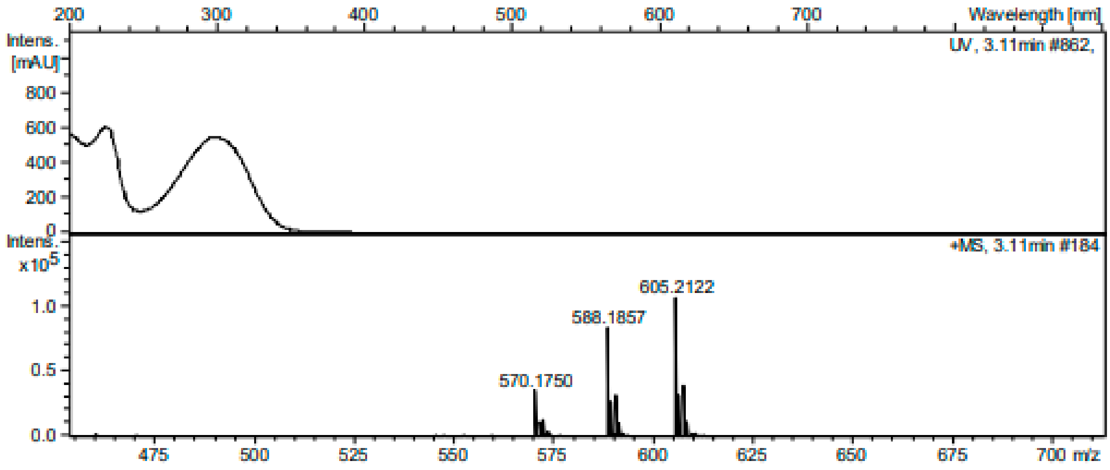

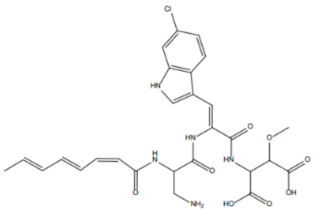

Colibrimycin A2, $C_{27}H_{28}ClN_5O_7$:

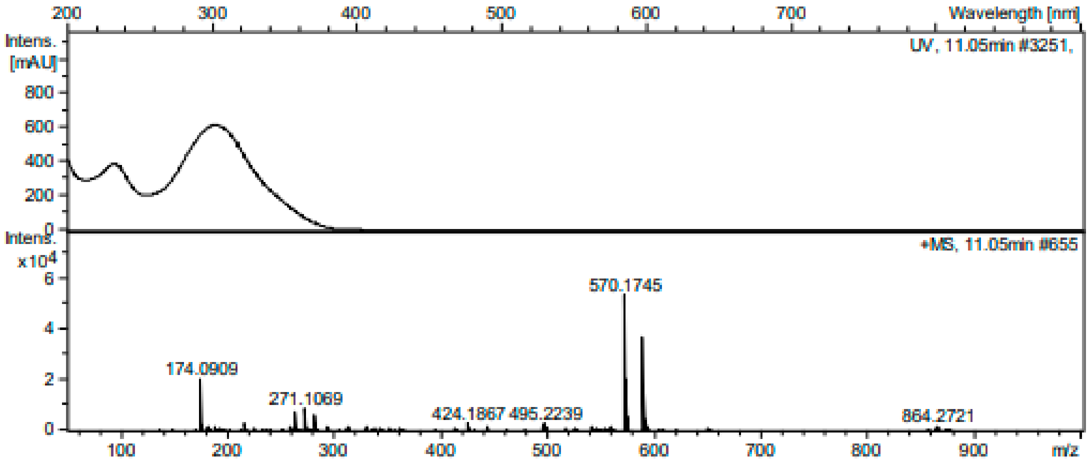

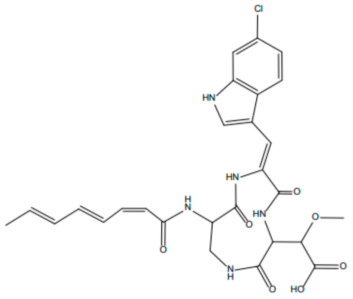

Colibrimycin A3, $C_{27}H_{28}ClN_5O_7$:

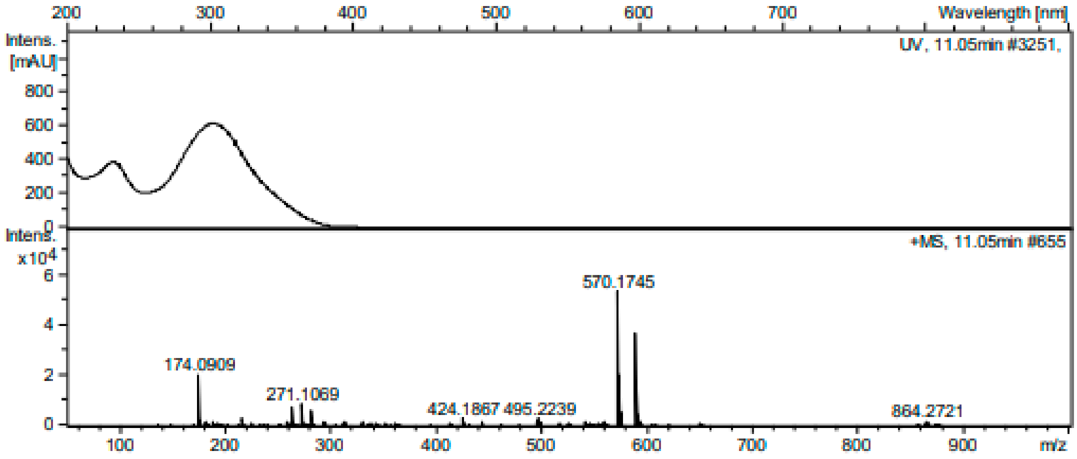

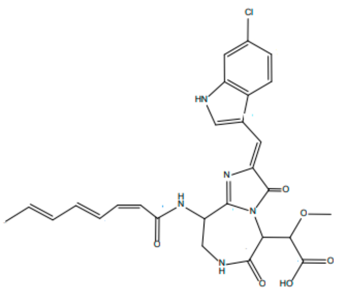

Colibrimycin A5, $C_{22}H_{25}ClN_4O_4$:

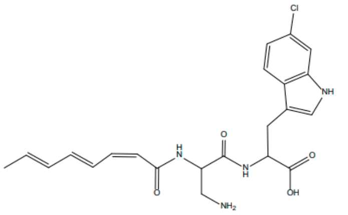

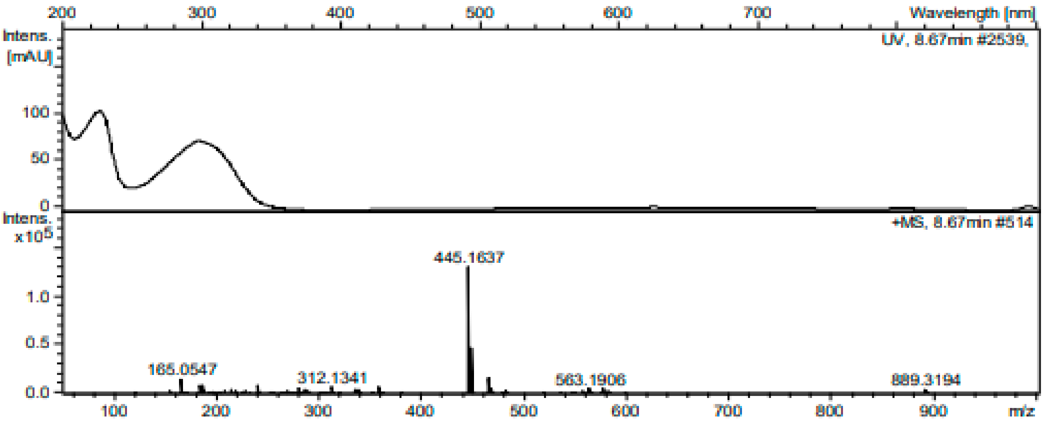

Colibrimycin C3, $C_{26}H_{29}ClN_4O_6$:

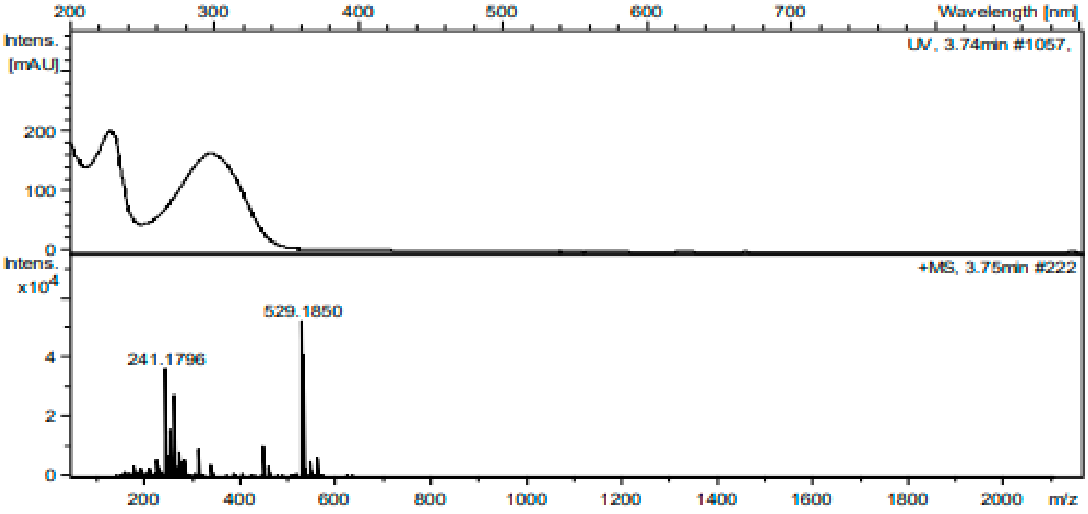

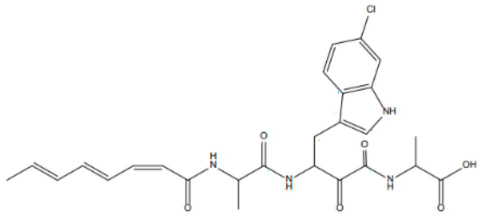

Colibrimycin C4, $C_{23}H_{28}ClN_3O_4$:

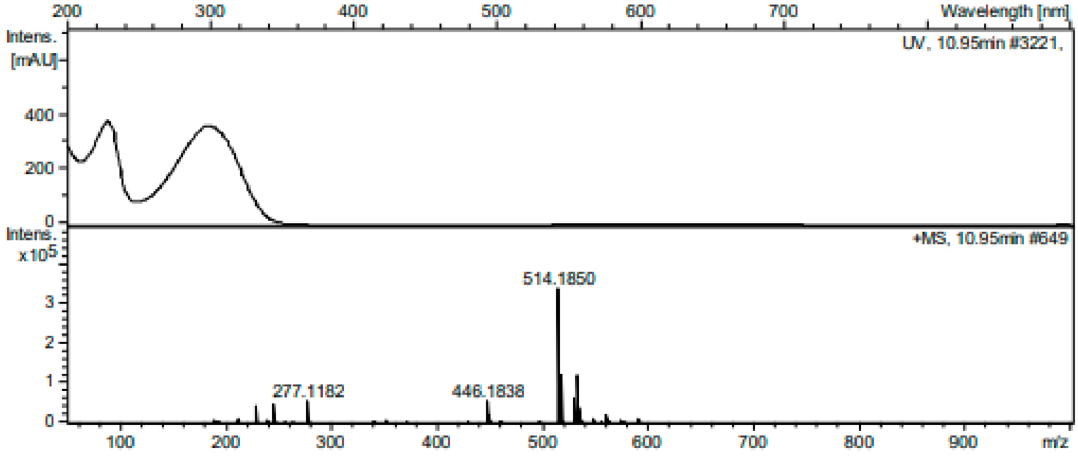

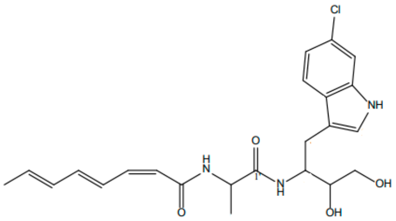

Colibrimycin C6, $C_{22}H_{24}ClN_3O_4$:

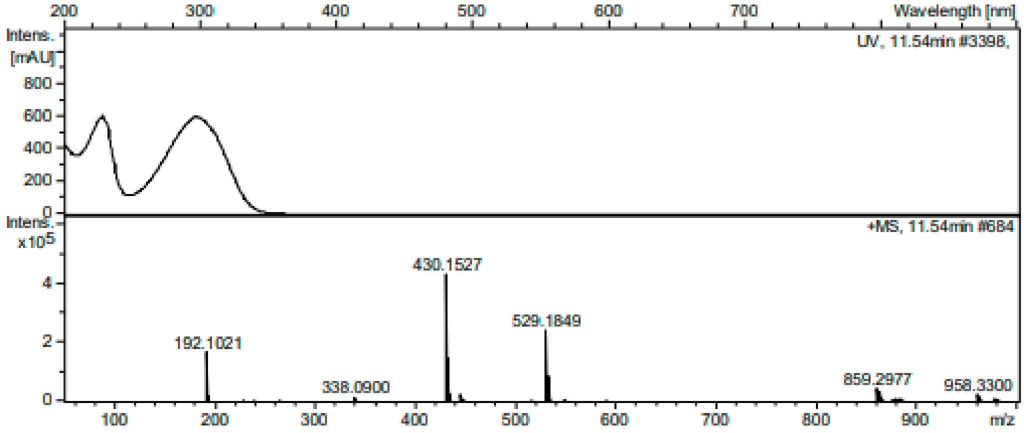

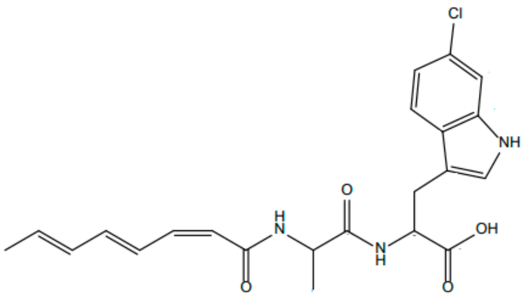

CS149:

Collismycin C, $C_{13}H_{13}N_3O_2S$:

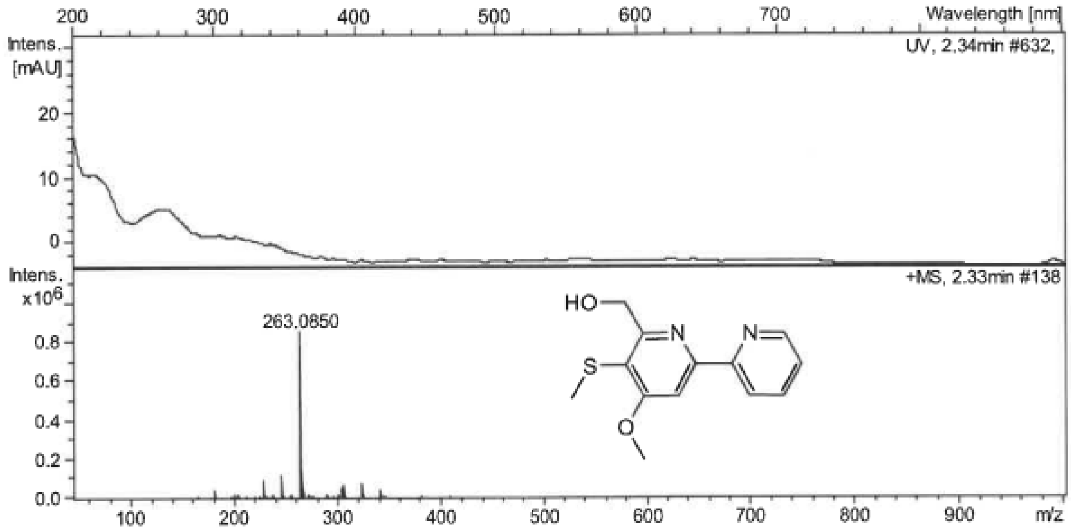

CS159:

Inthomycin, $C_{16}H_{22}N_2O_3$:

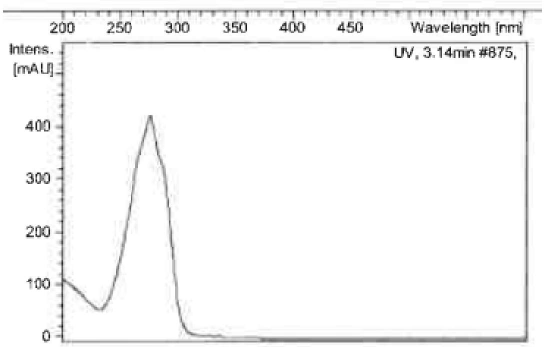

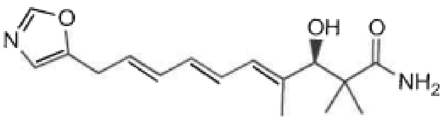

Undecylprodigiosin, $C_{25}H_{35}N_3O$:

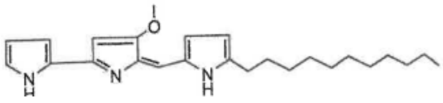

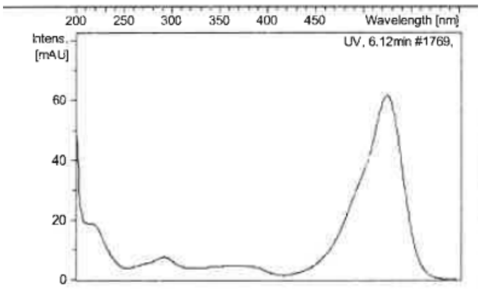

CS207:

3-(2-Hydroxyethyl)-6-prenylindole, $C_{15}H_{19}NO$

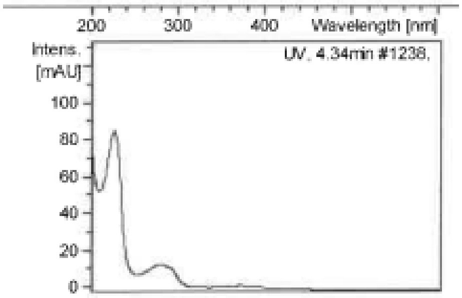

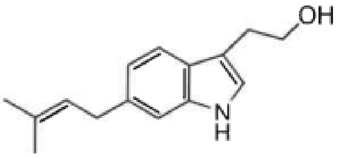

3-Cyanomethyl-6-prenylindole, $C_{15}H_{16}N_2$

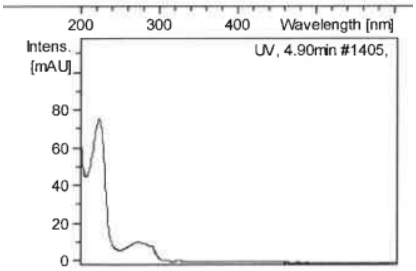

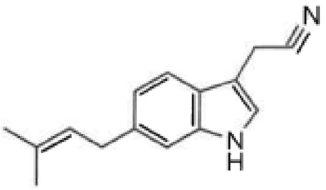

CS227:

2-Aminobenzoic acid, $C_7H_7NO_2$:

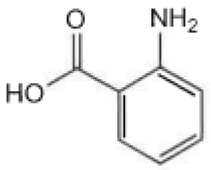

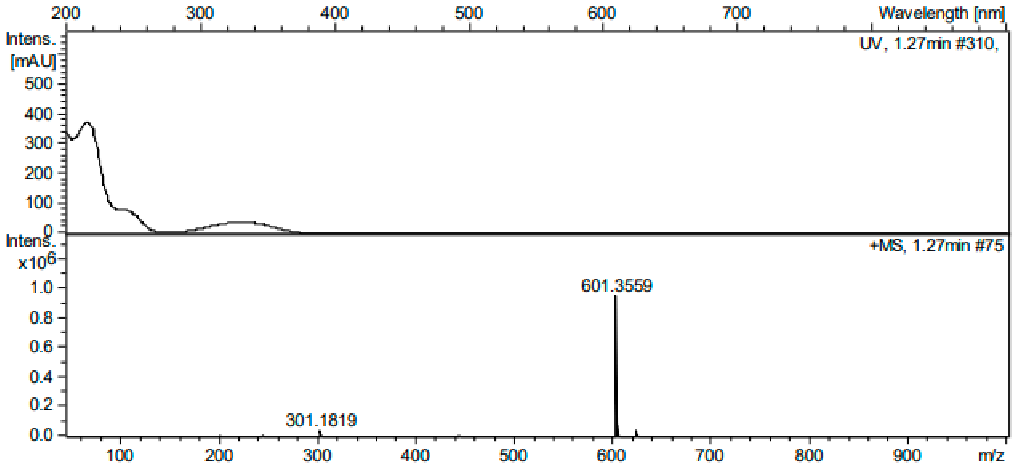

Suguramide A, $C_{48}H_{81}N_9O_8$:

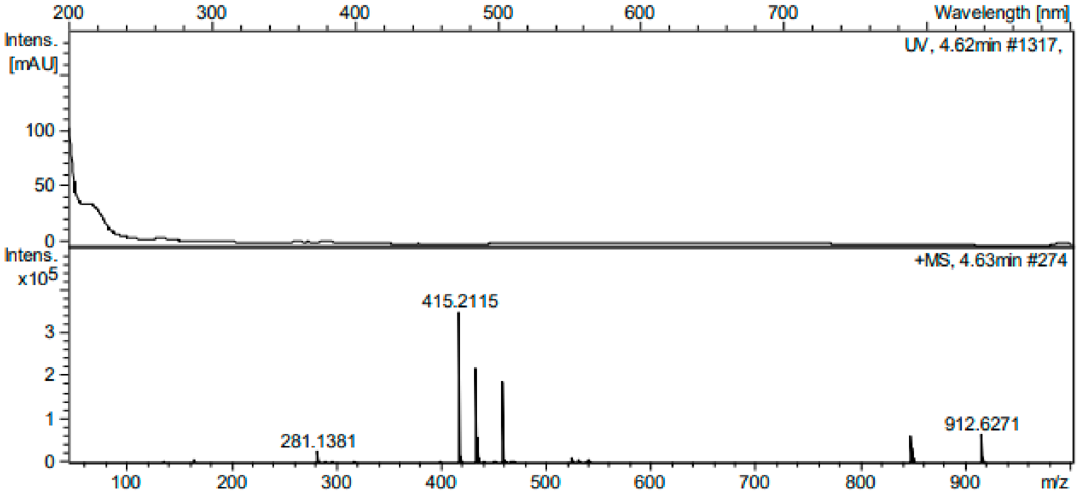

Several strains:

Coproporphyrin, $C_{36}H_{38}N_4O_8$

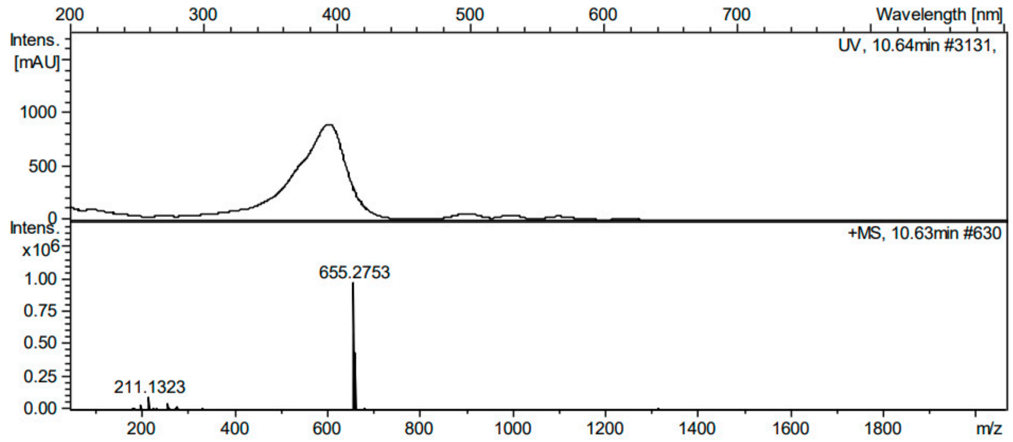

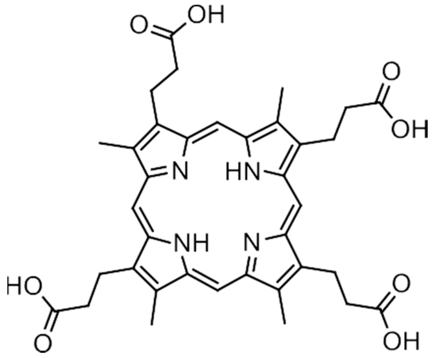

Table S4: Transcription Factor Binding Sites (TFBS) involved in this work find with strong confidence in the chromosome of each strain using AntiSMASH 7.0.

Strain	Cluster	Cluster type and MiBiG identification (over 65% similarity)	TFBS
CS014	1.22	Terpene (Geosmin 100%)	AbrC3
	1.23	Butyrolactone	AbrC3
	4.1	NRPS/PKS-I	AbrC3
CS057	1.7	PKS-I	AbrC3
	1.13	PKS-II/oligosaccharide/NRPS (warkmycin CS1/warkmycin CS2 97%)	AbrC3
CS065a	1.10	NRPS/NRPS-like	AbrC3
	1.15	nucleoside	AbrC3
	1.25	terpene	AbrC3
CS081a	1.3	Butyrolactone/NRPS	AbrC3
	4.7	PKS-II/terpene (Spore pigment 83%)	AbrC3
CS090a	1.7	Lanthipeptide –II/ Lanthipeptide -III	AbrC3
	1.15	Lanthipeptide –III (AmfS 100%)	AbrC3
	1.26	PKS-III/NRPS	AfsR
	1.28	NRPS/PKS-I/ Thiopeptide/ Linear azol(in)e-containing peptides/ RRE-element containing cluster	AbrC3
	2.1	Linear azol(in)e-containing peptides	AbrC3
	2.3	Terpene (Geosmin 100%)	AbrC3
CS113	1.4	RiPP-like	AbrC3
	1.19	NRPS-independent, IucA/IucC-like siderophores (Desferrioxamine 83%)	AbrC3
	1.22	PKS-II/PKS-like/oligosaccharide (Cervimycin 90%)	AbrC3
	1.25	Terpene	AfsR
	1.26	Butyrolactone/PKS-III (Germicidin 100%)	AfsR
			BldD
			AbrC3
CS131	1.2	Butyrolactone	AbrC3
	1.5	PKS-III (Naringenin 100%)	AbrC3
	1.6	NRPS-like/other (Actinomycin 89%)	AbrC3
	1.16	Lassopeptide (Stlassin 79%)	AbrC3
	1.25	Lanthipeptide -I	AfsR
	1.32	Terpene/PKS-I/NRPS (Isorenieratene 100%)	AbrC3
	1.37	NRPS/PKS-I	AbrC3
CS147	1.2	Butyrolactone	AbrC3
	1.5	PKS-III (Naringenin 100%)	AbrC3
	1.13	Phosphonate	AbrC3

Strain	Cluster	Cluster type and MiBiG identification (over 65% similarity)	TFBS
	1.31	NRPS/PKS-I	AbrC3
	1.32	Lassopeptide	AbrC3
			AfsR
CS149	1.12	Betalactone/Furan/Butyrolactone	AfsR
	1.13	Oligosaccharide/ Linear azol(in)e-containing peptides/ RRE-element containing cluster	AfsR
	1.31	NRPS/PKS-I	AbrC3
CS159	1.3	PKS-I/ PKS-like (arsono-polyketide 80%)	AbrC3
	1.10	NRP-metallophore/NRPS/PKS-I/NRPS-like/prodigiosin (Undecylprodigiosin 100%)	AbrC3
	1.18	PKS-II	AbrC3
	1.19	NI-siderophore (Desferroxiamin 83%)	AbrC3
	1.25	Indole (5-dimethylallylindole-3-acetonitrile 100%)	AfsR
CS207	1.2	PKS-II (Spore pigment 66%)	AbrC3
	1.9	Indole (5-dimethylallylindole-3-acetonitrile 100%)	AfsR
	1.12	PKS-II/Butyrolactone (Fluostatins M-Q 65%)	AfsR
	2.8	Lanthipeptide -III	AfsR
CS227	1.1	NRPS/NRPS-like	AfsR
	1.14	NRPS	AbrC3
	1.16	NRPS-independent, IucA/IucC-like siderophores (Desferroxiamin B 100%)	AbrC3

Table S5: TTA codons find in the chromosome of each strain using AntiSMASH 7.0.

Strain	Cluster	Type of cluster and MiBiG identification (over 65% similarity)
CS014	1.1	NRPS-like/NRPS
	1.3	PKS-I/NRPS (SGR PTMs 100%)
	1.4	Terpene (hopene 69%)
	1.5	NRPS (Holomycin 92%)
	1.6	NRPS/ PKS-I (Collismycin A 77%)
	1.8	Oligosacaride/PKS-I (Sipanmycin 100%)
	1.10	Terpene
	1.11	Lanthipeptide-III (AmfS 100%)
	1.16	Lanthipeptide-III/ Lanthipeptide-II
	1.20	PKS-III (Naringenin 100%)
	1.22	Terpene (Geosmin 100%)
	1.24	NRPS-like
	2.1	NRPS/ PKS-I
	2.4	Thiopeptide/ Linear azol(in)e-containing peptides
	2.5	NRPS/PKS-III
	4.1	NRPS/PKS-I
CS057	1.1	NRPS/PKS-I
	1.2	NRPS-like/lanthipeptide- IV/ transAT-PKS (Cycloheximide 94%)
	1.3	NRPS/PKS-III

Strain	Cluster	Type of cluster and MiBiG identification (over 65% similarity)
	1.6	NRPS/PKS-I
	1.7	PKS-I
	1.8	RiPP-like
	1.9	NRPS/NRPS-like (SGR PTMs 100%)
	1.11	Terpene (Hopene 69%)
	1.13	PKS-II/oligosaccharide/NRPS (warkmycin CS1/warkmycin CS2 97%)
	1.15	Terpene
	1.16	Lanthipeptide-III (AmfS 100%)
	1.20	Ectoine/butyrolactone/ladderane/arylpolyene/NRPS/PKS-I (Skyllamycins 97%)
	1.23	Lanthipeptide-III/ Lanthipeptide-II
	1.28	Thiopeptide/ Linear azol(in)e-containing peptides
	1.29	PKS-III (Narigenin 100%)
	1.31	Terpene (Geosmin 100%)
	1.32	Butyrolactone
CS065a	1.6	NRPS/PKS-I (Malthophilin 100%)
	1.7	NRPS
	1.8	Terpene (hopene 69%)
	1.9	arylpolyene/NRPS-like/ectoine
	1.10	NRPS-like/ NRPS
	1.12	Terpene
	1.13	Lanthipeptide-III (Amfs 100%)
	1.15	Nucleoside
	1.17	NRPS/PKS-I
	1.19	NRP-metallophore/NRPS/PKS-like
	1.20	Thiopeptide/ Linear azol(in)e-containing peptides
	1.22	Lanthipeptide-III/ Lanthipeptide-II
	1.23	PKS-II/oligosaccharide (Chromomycin 100%)
	1.26	PKS-III
	2.2	Terpene (Geosmin 100%)
CS081a	1.3	Butyrolactone/NRPS
	1.4	PKS-II/oligosaccharide/PKS-like (Cosmomycin D 97%)
	1.5	Butyrolactone/furan
	1.9	NRPS
	2.1	Lassopeptide (Albusnodin 100%)
	4.1	Lanthipeptide-III
	4.3	NRPS
	4.5	Terpene (Geosmin 100%)
	4.6	NRP-metallophore/NRPS
	4.9	PKS-I
	5.1	Phenazide
CS090a	1.1	PKS-III (Naringenin 100%)
	1.2	NRPS-like/NRPS-betalactone
	1.3	NRPS-like/terpene (2-methylisoborneol 100%)
	1.6	PKS-II/oligosaccharide
	1.7	Lanthipeptide-II/ Lanthipeptide-III
	1.9	NRPS-like
	1.10	Thiopeptide/ Linear azol(in)e-containing peptides
	1.12	PKS-I

Strain	Cluster	Type of cluster and MiBiG identification (over 65% similarity)
	1.15	Lanthipeptide-III (AmfS 100%)
	1.16	Terpene
	1.18	Butyrolactone
	1.19	Lanthipeptide-I
	1.20	Terpene (Hopene 69%)
	1.21	NRPS/PKS-I (Maltophilin 100%)
	1.23	NRPS
	1.24	NRPS/RiPP-like
	1.26	PKS-III/NRPS
	1.28	NRPS/PKS-I/thiopeptide/ Linear azol(in)e-containing peptides/ RRE-element containing cluster
	2.3	Terpene (Geosmin 100%)
	2.4	PKS-like/PKS-I/NRPS/NRP-metallophore/ transAT-PKS (Griseobactin 100%)
	4.1	PKS-III
	5.2	PKS-like
CS113	1.1	PKS-I/NRPS/NRP –metallophore/NRPS-like/other
	1.2	Other
	1.12	PKS-I/ NRPS-like/prodigiosin (Undecylprodigiosin 100%)
	1.14	NRPS
	1.16	Terpene (Albaflavenone 100%)
	1.26	Butyrolactone/ PKS-III (Germicidin 100%)
	1.27	Terpene (Isorenieratene (71%)
	1.28	Indole (5-dimethylallylindole-3-acetonitrile 100%)
CS131	1.1	NRPS-like
	1.3	Terpene (Geosmin 100%)
	1.6	NRPS-like/other (Actinomycin 89%)
	1.7	Terpene (Isorenieratene 87%)
	1.8	NRPS/ NRPS-like
	1.9	Terpene
	1.11	Lanthipeptide-II/ Lanthipeptide-III
	1.12	NRPS-independent, IucA/IucC-like siderophores (Desferroxiamin B 100%)
	1.14	NRPS-like
	1.15	NRPS
	1.16	Lassopeptide (Stlassin 79%)
	1.17	Lanthipeptide-II
	1.18	NRPS/Ladderane/arylpolyene/NRPS-like/Lassopeptide
	1.21	Lanthipeptide-III (AmfS 100%)
	1.23	Terpene
	1.25	Lanthipeptide-I
	1.26	NRPS (Holomycin 92%)
	1.29	Terpene (Hopene 69%)
	1.30	PKS-I/NRPS (SGR PTMs 100%)
	1.32	Terpene/PKS-I/NRPS (Isorenieratene 100%)
	1.35	thiopeptide/ Linear azol(in)e-containing peptides
	1.36	PKS-III/NRPS
	1.38	PKS-I (Stambomycins 96%)
CS147	1.1	NRPS-like

Strain	Cluster	Type of cluster and MiBiG identification (over 65% similarity)
	1.3	Terpene (Geosmin 100%)
	1.4	NRP-metallopore, NRPS, NRPS-like (Griseobactin 100%)
	1.5	PKS-III (Naringenin 100%)
	1.6	Arylpolyene
	1.7	Terpene (Isorenieratene 87%)
	1.10	Lanthipeptide-II/ Lanthipeptide-III
	1.13	Phosphonate
	1.16	Ladderane/NRPS (Colibrimycin (75%)
	1.17	PKS-I/ RRE-element containing cluster (Vicenistatin 100%)
	1.19	Lanthipeptide-III (AmfS 100%)
	1.20	Terpene
	1.22	NRPS (Holomycin 92%)
	1.24	Terpene (Hopene 69%)
	1.25	NRPS/PKS-I/RiPP-like (SGR PTMs 100%)
	1.26	Terpene/ NRPS/PKS-I (Isorenieratene 100%)
	1.27	RiPP-like (Streptamidine 66%)
	1.32	Lassopeptide
CS149	1.1	NRPS-like
	1.3	Terpene (Geosmin 100%)
	1.5	Terpene (Isorenieratene 100%)
	1.6	PKS-III (Narigenin 100%)
	1.10	Lanthipeptide-II/ Lanthipeptide-III
	1.12	Betalactone/Furan/Butyrolactone
	1.16	Lanthipeptide-III (AmfS 100%)
	1.17	NRPS/ NRPS-like
	1.18	Terpene
	1.20	Oligosaccharide/PKS-I (Sipanmycin 100%)
	1.21	NRPS/PKS-I (Collismycin A 70%)
	1.22	NRPS (Holomycin 92%)
	1.25	NRPS/PKS-I/RiPP-like (SGR PTMs 100%)
	1.26	NRPS/PKS-I
	1.29	thiopeptide/ Linear azol(in)e-containing peptides
CS159	1.5	Trans-AT PKS/NRPS/NRPS-like (Inthomycin B 100%)
	1.8	Betalactone
	1.10	NRP-metallophore/NRPS/PKS-I/NRPS-like/prodigiosin (Undecylprodigiosin 100%)
	1.11	N-siderophore
	1.12	PKS-II (Spore pigment 66%)
	1.13	Terpene (Albaflavenone 100%)
	1.16	Other
	1.17	NRPS (Sarpeptin 91%)
	1.18	PKS-II
	1.20	Melanin
	1.22	PKS-III (Flaviolin/1,3,6,8-tetrahydroxynaphthalene 100%)
	1.23	PKS-III (Germicidin 100%)
	1.25	Indole (5-dimethylallylindole-3-acetonitrile 100%)
	1.26	Terpene (2-methylisoborneol 100%)
	2.1	PKS-II/Butyrolactone
CS207	1.2	PKS-II (Spore pigment 66%)

Strain	Cluster	Type of cluster and MiBiG identification (over 65% similarity)
	1.6	PKS-III (Flaviolin/1,3,6,8-tetrahydroxynaphthalene 100%)
	1.7	NRPS-like (Streptothricin 95%)
	1.8	terpene
	1.9	Índole (5-dimethylallylindole-3-acetonitrile 100%)
	1.10	terpene
	1.11	Indole (7-prenylisatin 83%)
	1.12	PKS-II/Butyrolactone (Fluostatins M-Q 65%)
	2.22	NRPS (CDA 72%)
CS227	1.1	NRPS-like/ NRPS
	1.4	NRPS/ PKS-I (SGR PTMs 100%)
	1.7	PKS-I/ Terpene
	1.9	Terpene (Geosmin 100%)
	1.14	NRPS
	1.20	PKS-I/NRPS/Lanthipeptide-II/transAT-PKS/NRPS-like (Antimycin 100%)

References

1. Saranaruk, P.; Kariya, R.; Sittithumcharee, G.; Boueroy, P.; Boonmars, T.; Sawanyawisuth, K.; Wongkham, C.; Wongkham, S.; Okada, S.; Vaeteewoottacharn, K. Chromomycin A3 suppresses cholangiocarcinoma growth by induction of S phase cell cycle arrest and suppression of Sp1-related anti-apoptotic proteins. *Int J Mol Med* **2020**, *45*, 1005-1016.
2. Ding, Y.; Li, Y.; Li, Z.; Zhang, J.; Lu, C.; Wang, H.; Shen, Y.; Du, L. Alteramide B is a microtubule antagonist of inhibiting Candida albicans. *Biochim Biophys Acta* **2016**, *1860*, 2097-2106.
3. Morioka, H.; Etoh, Y.; Horino, I.; Takezawa, M.; Ando, T.; Hirayama, K.; Kano, H.; Shibai, H. Production and Isolation of Cosmomycins A, B, C and D: New Differentiation Inducers of Friend Cell F5-5. *Agric Biol Chem* **1985**, *49*, 1951-1958.
4. Jakobi, M.; Winkelmann, G.; Kaiser, D.; Kempler, C.; Jung, G.; Berg, G.; Bahl, H. Maltophilin: a new antifungal compound produced by Stenotrophomonas maltophilia R3089. *J Antibiot (Tokyo)* **1996**, *49*, 1101-1104.
5. Doungsoongnuen, S.; Worachartcheewan, A.; Pingaew, R.; Suksrichavalit, T.; Prachayasittikul, S.; Ruchirawat, S.; Prachayasittikul, V. Investigation on biological activities of anthranilic acid sulfonamide analogs. *EXCLI J* **2011**, *10*, 155-161.
6. Aoki, Y.; Yoshida, Y.; Yoshida, M.; Kawaide, H.; Abe, H.; Natsume, M. Anthranilic Acid, a Spore Germination Inhibitor of Phytopathogenic *Streptomyces* sp. B-9-1 Causing Root Tumor of Melon. *Actinomycetologica* **2015**, *19*, 48.
7. Banerjee, M.; Behera, C.C.; Pradhan, G.C.; Afzal-Azam, M.; Sahu, S.K. Synthesis and Biological Evaluation of some Anthranilic Acid and 2-Phenylquinazoline-4(3H)-one Analogues. *S Afr J Chem* **2009**, *62*, 134.
8. Petersen, F.; Zahner, H.; Metzger, J.W.; Freund, S.; Hummel, R.P. Germicidin, an autoregulative germination inhibitor of Streptomyces viridochromogenes NRRL B-1551. *J Antibiot (Tokyo)* **1993**, *46*, 1126-1138.
9. Choi, G.; Ryu, M.J.; Park, Y.G.; Nam, S.J. Antioxidant Activity of Germicidin A and B Isolated from the Marine-Derived *Streptomyces sp*. SCS525. *J Mar Biol Oceanogr* **2019**, *8*, 1.
10. Hou, S.; Zhang, M.; Wang, H.; Zhang, Y. Biosynthesis Gene Cluster and Oxazole Ring Formation Enzyme for Inthomycins in Streptomyces sp. Strain SYP-A7193. *Appl Environ Microbiol* **2020**, *86*, e01388-20. doi: 10.1128/AEM.01388-20. Print 2020 Oct 1.
11. Ramesh, C.; Anwesh, M.; Vinithkumar, N.V.; Kirubagaran, R.; Dufosse, L. Complete Genome Analysis of Undecylprodigiosin Pigment Biosynthesizing Marine Streptomyces Species

Displaying Potential Bioactive Applications. *Microorganisms* **2021**, *9*, 2249. doi: 10.3390/microorganisms9112249.
